# Role of the Substrate Specificity-Defining Residues of Human SIRT5 in Modulating the Structural Stability and Inhibitory Features of the Enzyme

**DOI:** 10.1371/journal.pone.0152467

**Published:** 2016-03-29

**Authors:** Junru Yu, Manas Haldar, Sanku Mallik, D. K. Srivastava

**Affiliations:** 1 Department of Chemistry and Biochemistry, North Dakota State University, Fargo, North Dakota, United States; 2 Department of Pharmaceutical Sciences, North Dakota State University, Fargo, North Dakota, United States; University of Gdansk, POLAND

## Abstract

Sirtuins are emerging as the key regulators of metabolism and aging, and their potential activators and inhibitors are being explored as therapeutics for improving health and treating associated diseases. Despite the global structural similarity among all seven isoforms of sirtuins (of which most of them catalyze the deacetylation reaction), SIRT5 is the only isoform that catalyzes the cleavage of negatively charged acylated substrates, and the latter feature appears to be encoded by the presence of Tyr102 and Arg105 residues at the active site pocket of the enzyme. To determine the contributions of the above residues in SIRT5 (vis a vis the corresponding residues of SIRT1) on substrate selectivity, inhibition by EX527 and nicotinamide, secondary structural features and thermal stability of the enzymes, we created single and double mutations (viz. Y102A, R105l, and Y102A/R105I) in SIRT5. The kinetic data revealed that while Y102A mutant enzyme catalyzed both deacetylation and desuccinylation reactions with comparable efficiencies, R105I and Y102A/R105I mutant enzymes favored the deacetylase reaction. Like SIRT1, the nicotinamide inhibition of SIRT5 double mutant (Y102A/R105I) exhibited the mixed non-competitive behavior. On the other hand, the desuccinylation reaction of both wild-type and Y102A mutant enzymes conformed to the competitive inhibition model. The inhibitory potency of EX527 progressively increased from Y102A, R105I, to Y102A/R105 mutant enzymes in SIRT5, but it did not reach to the level obtained with SIRT1. The CD spectroscopic data for the wild-type and mutant enzymes revealed changes in the secondary structural features of the enzymes, and such changes were more pronounced on examining their thermal denaturation patterns. A cumulative account of our experimental data reveal mutual cooperation between Y102 and R105 residues in promoting the desuccinylation versus deacetylation reaction in SIRT5, and the overall catalytic feature of the enzyme is manifested via the mutation induced modulation in the protein structure.

## Introduction

Silent information regulator 2 (Sir 2 or sirtuin) belongs to an ancient family of proteins which are highly conserved in various organisms ranging from bacteria to humans [1]. These proteins were originally identified as gene silencing regulators in budding yeast, and subsequently, they were categorized as the Class III histone deacetylases (HDACs), exhibiting the nicotinamide adenine dinucleotide (NAD^+^) dependent lysine-deacetylase activities [2]. Due to their involvement in a variety of biological processes such as gene transcription, DNA repair, apoptosis, metabolism and aging [1, 3, 4], sirtuins have been considered as the high priority drug targets of life extension as well as for controlling the age related diseases [4, 5].

There are seven isoforms of human sirtuins (SIRT1-SIRT7) which are categorized on the basis of their conserved catalytic cores. Although all sirtuins have their primary localization sites in different subcellular compartments (e.g., SIRT1, SIRT6 and SIRT7 in nucleus, SIRT2 in cytoplasm, SIRT3, SIRT4 and SIRT5 in mitochondria), they tend to shuttle from one subcellular compartment to the other depending on the physiological/pathological conditions of cells [6–9]. Although most sirtuins primarily catalyze the deacetylation of acetylated lysine peptide substrates, the substrate specificities of other sirtuins are somewhat different from each other. Feldman et al. recently demonstrated that SIRT1-SIRT6 all possess the potential to catalyze the deacylation reactions of fatty acyl-lysine conjugates in peptides/proteins [10, 11]. Besides, SIRT4 and SIRT6 have been found to carry out the ADP ribosyltransferase reaction in the absence of acylated substrates [12, 13]. More recently, SIRT5 has been found to possess robust desuccinylase, demalonylase and deglutarylase activities but only weak deacylase activity [14, 15]. The above substrate specificity of SIRT5 has been demonstrated to be physiologically relevant as many metabolic enzymes exist in succinylated/malonylated forms and their removal results in the regulation (activation/inhibition) of the associated enzymes [14, 16, 17]. The uniqueness of the above SIRT5 mediated reaction has been shown to be encoded in the presence of Y102 and R105 residues at the active site pocket of the enzyme [14].

**Fig 1:**
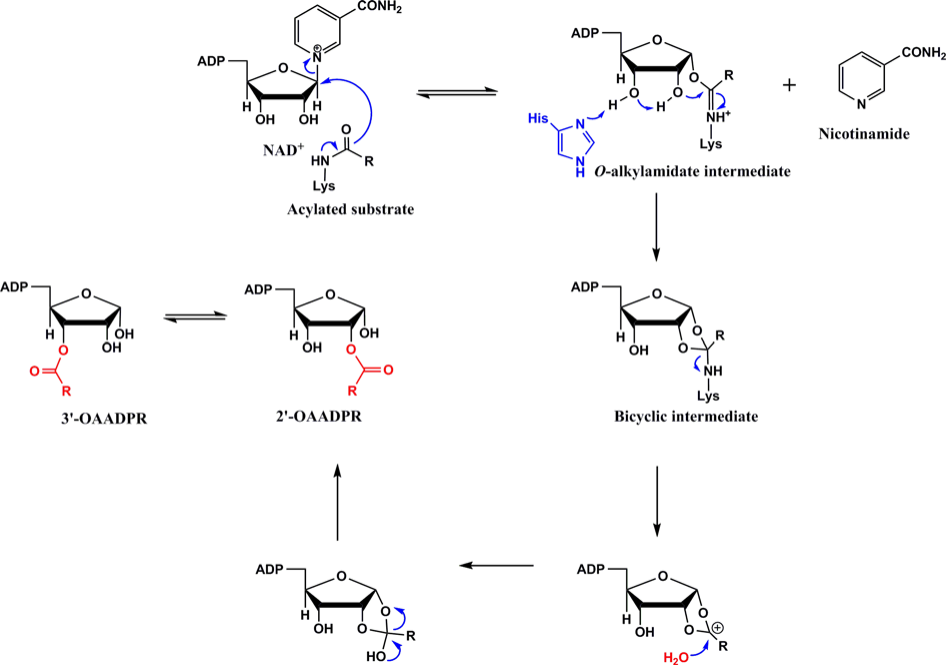
Schematic Representation of the Sirtuin Catalyzed Reaction Mechanism: The sirtuin catalyzed reaction proceeds via the nucleophilic attack of the carbonyl oxygen of the acylated substrate to the C1’ ribose of NAD^+^, resulting in the cleavage of the nicotinamide moiety of NAD^+^ and formation of C1′-*O*-alkylimidate intermediate. The alkylimidate intermediate undergoes the cyclization reaction followed by hydrolysis, generating the deacylated product and 2′-*O*-acyl-ADP-ribose (OAADPr) species. The latter is slowly isomerized (non-enzymatically) to form 3′-OAADPr.

As summarized in Fig 1, the catalytic mechanism of sirtuin reaction proceeds via the nucleophilic attack of the carbonyl oxygen of the acylated substrate to the C1’ ribose of NAD^+^, resulting in the cleavage of the nicotinamide moiety of NAD^+^ with concomitant formation of C1’-O-alkylimidate intermediate [18]. In the presence of high concentration of nicotinamide, the above intermediate reverses back to the original reacting species (formally referred to as the “base exchange” reaction), resulting in the inhibition of several sirtuin isoforms [19, 20]. The *O*-alkylimidate intermediate undergoes the cyclization reaction followed by the release of the deacylated product and generation of 2′-*O*-acyl-ADP-ribose (OAADPr) species. The latter is slowly (non-enzymatically) isomerized to form 3′-OAADPr as the final reaction product. The levels of NAD^+^, nicotinamide and 2′/3′-OAADPr modulate a wide range of physiological processes, which are manifested in the sirtuin mediated life extension and alleviation of the age-related diseases [21–23]. From the kinetic point of view, except for SIRT6, all sirtuins adhere to the compulsory order mechanism in which the binding of substrate precedes the binding of NAD^+^; in case of SIRT6, the binding of substrate and NAD^+^ occurs in a random order fashion [24–26].

**Fig 2:**
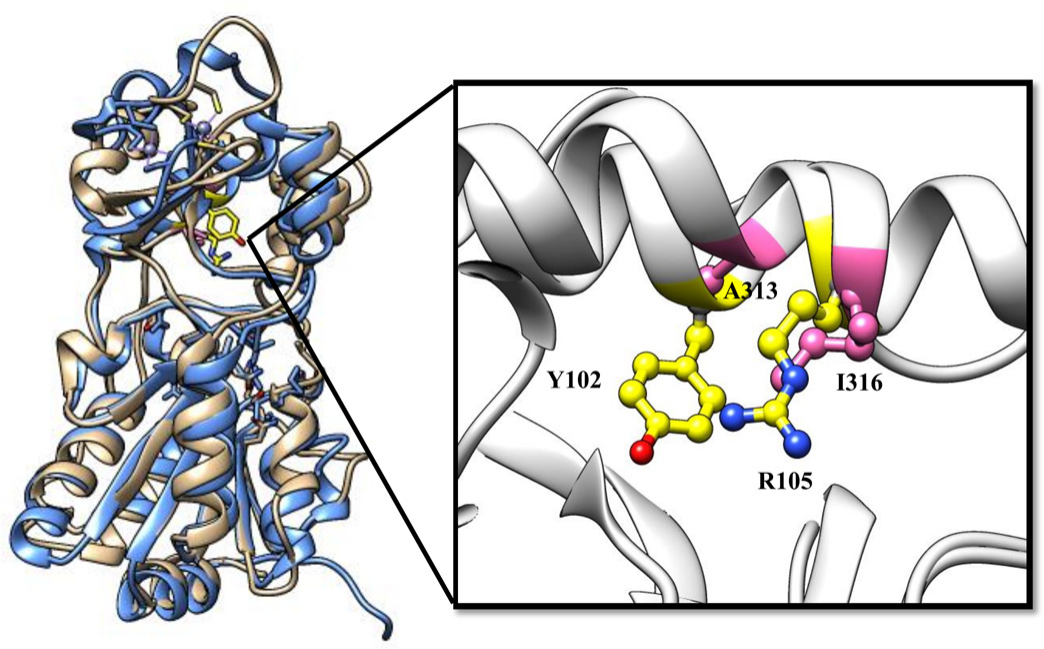
Comparative Structural Features in the Catalytic Domains of SIRT5 and SIRT1. (A) The superimposition (ribbon diagrams) of the catalytic domains of human SIRT5 (blue) and SIRT1 (tan). (B) A close view at the active site pockets of SIRT5 and SIRT1. The side chains of Y102 and R105I (yellow) in SIRT5 and of A313 and I316 (pink) in SIRT1 are presented as ball-and-stick models.

We recently became interested in the mechanistic aspects of the SIRT1 and SIRT5 catalyzed reactions from the point of view of designing isozyme selective activators and inhibitors as potential drugs [27–36]. In pursuit of this objective, we sought to probe the mechanistic origin of the substrate selectivity between SIRT1 and SIRT5. While SIRT1 primarily catalyzes the deacetylation reaction (of acetylated lysine residues) in selected proteins, SIRT5 preferentially cleaves the negatively charged acyl moieties (viz., succinyl-, malonyl-, glutaryl-) from their lysine conjugates in enzymes and proteins [14, 15, 37]. The X-ray crystallographic data revealed that the above substrate specificity is basically encoded in the substitution of A313 and I316 of SIRT1 by Y102 and R105 in SIRT5, respectively. Fig 2 shows the alignment of the catalytic subunits of SIRT1 and SIRT5, and the relative positions of the above residues are shown in the enlarged view of the active site pockets. Note a marked correspondence between the secondary structural features as well as the relative positions of the substrate specificity-defining residues in SIRT1 and SIRT5. In view of the above structural similarity/difference between the two enzymes, we enquired whether the desuccinylase activity of SIRT5 could be reverted back to the deacetylase activity (as given by SIRT1) by replacing Y102 and R105 residues of SIRT5 by Ala and Ile (found in SIRT1), respectively, via site directed mutagenesis. Interestingly, we observed that the above mutations not only altered the substrate specificity of SIRT5, but they also altered the secondary structural features, thermal stability as well as sensitivity of the enzyme to nicotinamide and EX527 inhibition. Our data clearly demonstrate that Y102 and R105 of SIRT5 are not only involved in stabilizing the negative charged acyl groups of the succinylated substrate at the active site of the enzyme, but also influence the secondary structural, kinetic, and inhibitory features of the enzyme. Such differences could not be conceived in the light of the X-ray crystallographic data of the enzyme [14, 38].

## Materials and Methods

### Materials

The plasmids containing the coding sequence of human SIRT1 (pOTB7) and SIRT5 (pOTB7) were obtained from Open Biosystem (Huntsville, AL). The ligation independent cloning (LIC) vector, compatible with the E. coli expression, was a kind gift from Prof. Stephen P. Bottomley (Monash University, Australia). *Fluor-de-Lys*^*®*^ (KI-104) and deacetylated lysine-coumarin adduct (BML-KI142) were purchased from Enzo Life Sciences Inc. (Plymouth Meeting, PA). Nicotinamide and isonicotinamide were purchased from Sigma (St. Louis, MO). EX527 was purchased from Cayman Chemical Company (Ann Arbor, MI). The succinylated substrate (Ac-SucLys-AMC) was synthesized following the protocol described by Andreas et al [39].

### Cloning, expression and purification of recombinant proteins

SIRT1 (193–747) and SIRT5 (51–301) were cloned into the pLIC-His expression vector as described previously [40]. The wild type SIRT5 plasmid was used as a template for the site-directed mutagenesis, using the QuikChange II site-directed mutagenesis kit (Agilent Technologies) to create three SIRT5 mutants, Y102A, R105I and Y102A/R105I. The mutations were confirmed by sequencing the resultant plasmids (Mclab, San Francisco, CA). The plasmids were transformed into BL21(DE3) Star^®^ cells (Invitrogen), expressed and purified as described by Milne et. al. [41]. Soluble proteins of the cell lysate were purified by immobilized metal affinity chromatography using Histrap HP column (GE Healthcare). SIRT1 was further purified by HitrapQ HP ion exchange chromatography (GE Healthcare). The purified enzymes were subjected to the SDS-PAGE analysis and stored in 50 mM HEPES (pH 7.5), 100 mM NaCl, 10% glycerol and 1 mM TCEP at -70°C.

### Circular dichroism spectroscopy

Far-UV CD spectra (190–250 nm) were collected on a J-710 spectropolarimeter (Jasco, Tokyo, Japan) equipped with a Peltier temperature controller. The enzyme stocks were diluted to 10 μM in 5 mM Tris-HCl buffer, pH 8.0, containing 0.5% glycerol, 0.1 mM TCEP and 15 mM KCl, and transferred to a 1 mm path-length quartz cuvette for obtaining the CD spectra. The samples were subjected to the CD spectral acquisitions from 190 nm to 250 nm in triplicates and the spectra were averaged. The CD signals were converted to the mean residue ellipticity (MRE; *θ*) using Eq 1.

$$\theta =\frac{signal}{10\cdot l\cdot c}\cdot \frac{1}{r}$$ (1)

where “*signal*” is the amplitude of the raw CD signal; *l* is the path length (mm) of the cuvette; c is the protein concentration, and *r* is the number of amino acid residues in the protein.

The thermal unfolding of the enzymes was performed by monitoring the changes in the CD signal at 208 nm as a function of increasing temperature (maintained via the Peltier controller) from 12 to 95°C at a rate of 1°C min^-1^. The data were analyzed either by a single (Eq 2) or double transition (Eq 3) Boltzmann equations, respectively.

$$\theta =\frac{A_{1}-A_{2}}{1+e^{\left(x-x_{0}\right)/dx}}+A_{2}$$ (2)

where *x* is the temperature, A_1_ and A_2_ are the initial (base line) and final (upon completion of unfolding) ellipticities, respectively; *x*_0_ is the melting temperature (T_m_), and *dx* is the width of the thermal transition,

$$\theta =\frac{A_{0}-A_{1}}{1+e^{\left(x-x_{01}\right)/dx1}}+\frac{A_{1}-A_{2}}{1+e^{\left(x-x_{02}\right)/dx2}}+A_{2}$$ (3)

Where *x* is the temperature, A_0_, A_1_ and A_2_ are the initial, intermediate, and final baseline ellipticities for the two sequential (independent) thermal transitions, respectively, with *x*_01_ and *x*_02_ being the melting temperatures (T_m_) and *dx*1 and *dx*2 being the widths of the first and second thermal transitions, respectively.

### Bi-substrate kinetic mechanism of the sirtuin catalyzed reactions

The sirtuin catalyzed deacetylase and desuccinylase reactions were measured via the trypsin coupled assay system as described by Madsen et al [39]. The enzyme reactions were carried out at 25°C in 50 mM Tris-HCl assay buffer, pH 8.0, containing 137 mM NaCl, 2.7 mM KCl, 1 mM MgCl_2_, and 1 mg/mL bovine serum albumin. Since trypsin was used as a coupling enzyme, its concentration was optimized to ensure the emergence of minimal or negligible lag-phase prior to the onset of the steady state phase [42]. The bi-substrate kinetic experiments were performed by varying the concentrations of NAD^+^ (from 100 to 500 μM) and Ac-SucLys-AMC or *Fluor-de-lys*^*®*^ substrates (from 100 μM to 1200 μM) using 0.8 μM trypsin as the coupling enzyme. The steady state rates of the enzyme catalyzed reactions were derived from the time dependent changes in the fluorescence signal, and the data were analyzed by the ternary complex kinetic mechanism (Eq 4) via Grafit 4 software.

$$v=(\frac{V_{\text{max}}[A][B]}{K_{\text{a}}K_{\text{mb}}+K_{\text{ma}}[B]+K_{\text{mb}}[A]+[A][B]})$$ (4)

Where V_max_ is the maximum reaction velocity, K_a_ is the dissociation constant for the acylated substrates (A), and K_ma_ and K_mb_ are the Michaelis-Menten constants for acylated substrates and NAD^+^ (B), respectively.

### Nicotinamide inhibition studies

The nicotinamide inhibition of sirtuin catalyzed reactions was determined by performing the steady-state kinetic experiments in the absence and presence of nicotinamide as described above. The experiments were performed in the presence of either 100 μM *Fluor-de-lys*^*®*^ or Ac-SucLys-AMC substrate with varying concentrations of NAD^+^ and nicotinamide. The steady-state kinetic data were fitted globally by the competitive, mixed, and noncompetitive inhibition models using the DYNAFIT software based on the rapid-equilibrium approximation [43]. All the parameters were allowed to float except for the enzyme concentration. The software utilizes several model-discrimination criteria, including the second order Akaike Information Criterion (AIC) [44, 45], so that the best model out of the three different candidates could be identified.

### IC_50_ of EX527 during sirtuin catalysis

The IC_50_ values of EX527 for the sirtuin catalyzed reactions were measured using the fixed concentrations of substrates and NAD^+^. For the inhibition studies of SIRT1 catalysis, the experiment was performed in the presence of 100 μM *Fluor-de-lys*^*®*^ substrate, 500 μM NAD^+^, and varying concentration (from 0 μM to 40 μM) of EX527. For the inhibition studies of wild-type and SIRT5 mutants catalyzed reactions by EX527, the experiments were carried in the presence of 100 μM *Fluor-de-lys*^*®*^ substrate, 500 μM NAD^+^, and varying concentration (from 0 μM to 200 μM) of EX527. Other reaction conditions were the same as described above. The data were fitted by Eq 5 by Grafit 4.0.

$$y=\;\frac{Range}{1+\left(\frac{x}{{IC}_{50}}\right)s}+\text{Background}$$ (5)

Where *Range* is the fitted uninhibited value minus the *Background*, and *s* is the slope factor. The parameters x and y are the concentrations of EX527 and initial rates of the enzyme catalysis, respectively.

### Isothermal titration calorimetry (ITC)

Isothermal titration calorimetric experiment was performed in duplicates on a VP-ITC device (Microcal Inc., Northampton, MA). Prior to the titration experiment, both enzyme and inhibitor were thoroughly degassed under vacuum. The sample cell was filled with 1.8 mL (effective volume = 1.4 mL) of 20 μM Y102A/R105I mutant SIRT5 and 0 or 10 mM NAD^+^ in 50 mM HEPES buffer, pH 7.5, containing 100 mM NaCl, 10% glycerol and 1 mM TCEP. The content of the sample cell was titrated with 45 aliquots (4 μl each) of EX527 containing either 0 or 10 mM NAD^+^, prepared in the same buffer. The data were analyzed by the ITC data analysis module of Origin Software using the single-site binding model as described by Wiseman et al [46]. All the parameters were allowed to vary during the curve-fitting. The data analysis produced three parameters, viz. stoichiometry (n), association constant (K_a_), and the standard enthalpy change (ΔH°) for the binding of EX527 to SIRT5 double mutant (Y102A/R105I) in the presence of NAD^+^. The binding free energy (ΔG°) and the entropic (ΔS°) changes were calculated by Eqs 6 and 7, respectively

$$\Delta G{}^{\circ}\;=-RTlnK_{\text{a}}$$ (6)

$$\Delta S{}^{\circ}\;=\frac{\Delta H{}^{\circ}-\Delta G{}^{\circ}}{T}$$ (7)

## Results

### Site specific mutations in the active site pocket of SIRT5 to mimic SIRT1

Du et al. demonstrated that the presence of two amino acids, viz., Y102 and R105, at the active site pocket of SIRT5 changes the substrate specificity of the enzyme from carrying out normal deacetylation to desuccinylation/demalonylase reaction [14]. The structural alignment between SIRT5 and SIRT1 (Fig 2) reveals that the above amino acids are substituted by Ala and Ile in SIRT1, respectively. To ascertain whether the above difference in the amino acid substitutions is exclusively responsible for the difference in the substrate specificity between SIRT1 and SIRT5, we created two single (Y102A and R105I) and one double mutation (Y102A/R105I) in SIRT5 by site-directed mutagenesis, expressed the wild-type and mutant enzymes in E. *coli*, and purified them to homogeneity as described in the Materials and Methods section. We observed that the expression levels of all three mutant enzymes were comparable to that of the wild type SIRT5. It should be pointed out that although other groups [14, 47] have created mutations in the R105 residue of SIRT5 (to either Met or Leu) to assess its role in modulating the substrate specificity and nicotinamide inhibition of the enzyme, to the best of our knowledge, no systematic studies on the influences of Y102 and R105 mutations in SIRT5 (to mimic the amino acid residues found in SIRT1) on the structural-functional features of the enzymes have been performed prior to our studies presented herein.

### Contributions of Y102 and R105 residues on the secondary structural features and stability of SIRT5

**Fig 3:**
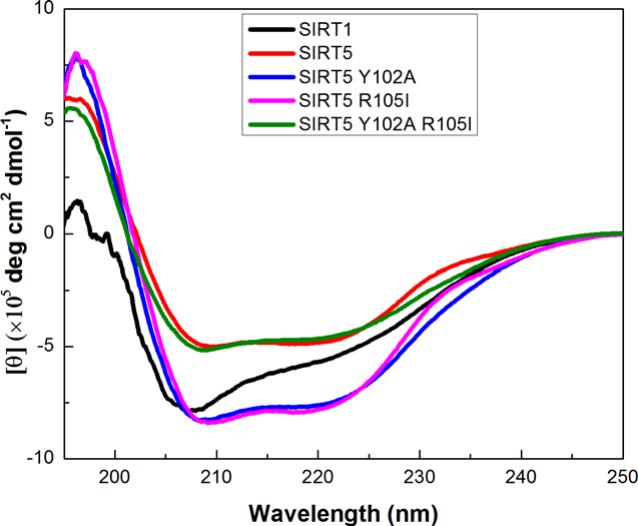
Circular Dichroim (CD) Spectra of SIRT5 Variants and SIRT1. The mean residue ellipticities of 10 μM enzymes as a function of wavelength are shown. The experiments were performed in 5 mM Tris-HCl buffer, pH 7.5 using a quartz cuvette of 1 mm path length.

In order to determine whether Y102A, R105I, and Y102A/R105I mutations in SIRT5 alter the secondary structural features of wild-type SIRT5, we performed the CD spectroscopic studies. Fig 3 shows the changes in the mean residue ellipticities (θ) of the enzymes as a function of wavelength. Note that except for SIRT1, the wild-type and mutant SIRT5 enzymes exhibit double minima at 208 and 222 nm, which suggest the predominance of α helices with minor (masked) contribution of β sheet structures. Although the elliptical features of both wild-type and mutant SIRT5 are qualitatively similar, there are noteworthy differences in the shapes as well as the amplitudes of the elliptical peaks among the above enzymes. Such differences are more apparent with SIRT5 containing the R105 mutation. In contrast, the CD spectrum of SIRT1 is dominated by the random coil structural features, presumably due to its extended N-terminal and C-terminal segments which are not highly organized.

**Fig 4:**
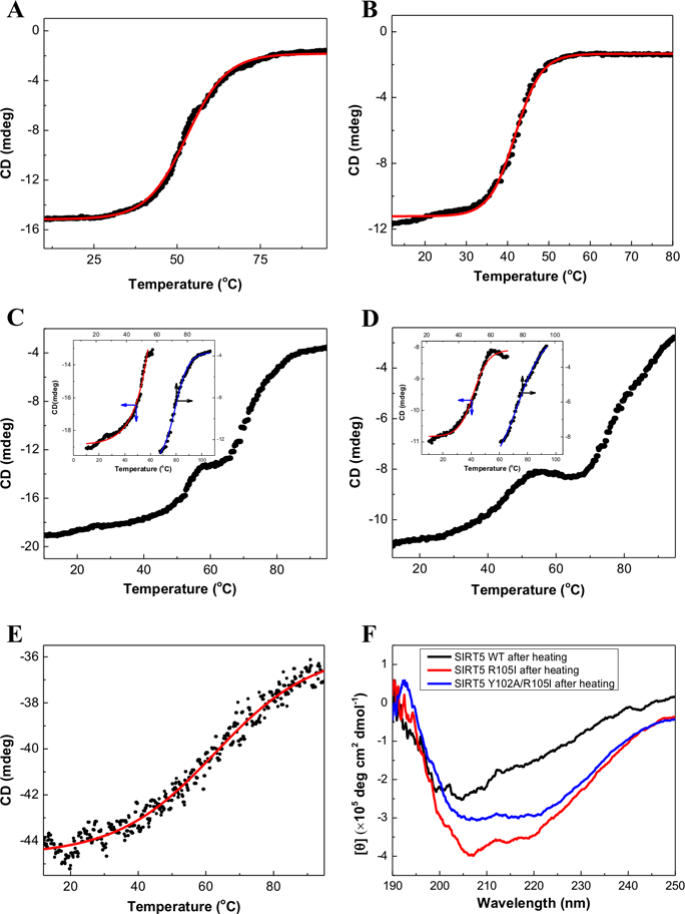
Thermal Denaturation Profiles of SIRT5 Variants and SIRT1. The ellipticity of (A) SIRT5 wild type, (B) Y102A, (C) R105I, (D) Y102A/R105I and (E) SIRT1were measured at 208 nm as a function of temperature. The solid smooth lines represent the best fit of the data by the single phase Boltzmann equation (Eq 2) to derive the T_m_ values. (F) The CD spectra of wild-type and mutant enzymes upon heating at their final melting temperatures followed by cooling at 25^**°**^**C**

To probe the influence of the above mutations on thermal stability of SIRT5, we determined the temperature dependent changes in the secondary structural features of the wild-type and mutant enzymes. As shown in Fig 4, the increase in temperature increases the ellipticities of the enzymes at 208 nm in a sigmoidal manner, resulting in the attainment of plateau at high temperatures. A casual perusal of the data of Fig 4 reveal that while wild-type SIRT5 and Y102A mutant enzyme exhibit a single stage melting transition (Fig 4A and 4B), R105I and Y102A/R105I mutant enzymes exhibit the two stage melting transitions (Fig 4C and 4D). When we performed the above experiment with wild-type SIRT1 (Fig 4E), the overall profile exhibited a single stage melting transition, similar to that observed with the wild-type SIRT5 (Fig 4A) and its Y102A mutant variant (Fig 4B). Depending on the nature of melting transition profiles of the enzymes, we attempted to analyze the data of Fig 4A to 4D either by single (Eq 2) or double (Eq 3) transition Boltzmann equations as described in the Materials and Methods section. Since the analyses of the data of Fig 4C and 4D by double transition Boltzmann equation (Eq 3) did not produce reliable results, recourse was made to analyze the individual melting transitions by the single transition Boltzmann equation (Eq 2). The derived T_m_ values (the temperature at which half of the proteins lost the secondary structural features) from the best fit of the data are summarized in Table 1. The data of Table 1 clearly shows that both wild-type SIRT1 and SIRT5 exhibit the single phase denaturation profile, but the T_m_ value of SIRT1 (62.7 ± 0.4°C) is about 10 degree higher than that of wild-type SIRT5 (52.9 ± 0.04°C). On the other hand, the T_m_ value of the wild-type SIRT5 is about 11 degree higher than that of the Y102A mutant (41.8 ± 0.03°C), suggesting that Y102A mutation destabilizes the enzyme structure. Interestingly, we noted that of two T_m_ values of R105I mutant of SIRT5, while the T_m_ value of the first transition phase (58.1 ± 1.0°C) resembles that of the wild-type SIRT5 (52.9 ± 0.04°C), the T_m_ value of the second transition phase (72 ± 0.1°C) is about 20 degree higher. On the other hand, while the T_m_ value of the first transition phase of Y102A/R105I double mutant (42.9 ± 0.1°C) resembles that of Y102A mutant enzyme (41.8 ± 0.03°C), the T_m_ value of the second transition phase (79.0 ± 0.1°C) is about 5 degree higher than that of the second phase of R105I mutant (72.0 ± 0.1°C). Clearly, Y102 and R105 residues exhibit differential effects on thermal stability of the enzyme.

We further investigated whether the fully denatured enzymes acquired the secondary structural features upon cooling to the room temperature (25^**°**^**C)**. To ascertain this, we heated the individual enzyme samples to their final melting temperatures, cooled them to room temperature (25^**°**^**C)**, and recorded their CD spectra (Fig 4F). To our surprise, we noted that while the above treatment of wild-type SIRT5 resulted in nearly complete loss of the secondary structural feature that of the R105I mutant showed modest secondary structure. On the other hand, the Y102A/R105I mutant enzyme showed the secondary structural feature similar to that of the wild-type enzyme. However, none of the heat treated (and subsequently cooled) samples showed any enzyme activity.

**Table 1:** Melting Temperatures of SIRT5 Variants as well as SIRT1.

	T_m_ (°C)
**SIRT5 wild type**	**52.9 ± 0.04**
**SIRT5 Y102A**	**41.8 ± 0.03**
**SIRT5 R105I**	**58.1 ± 1.0**
	**72.0 ± 0.1**
**SIRT5 Y102A/R105I**	**42.9 ± 0.1**
	**79.0 ± 0.1**
**SIRT1**	**62.7 ± 0.4**

### Bisubstrate kinetic studies for the wild-type and mutant enzyme catalyzed reactions

**Fig 5:**
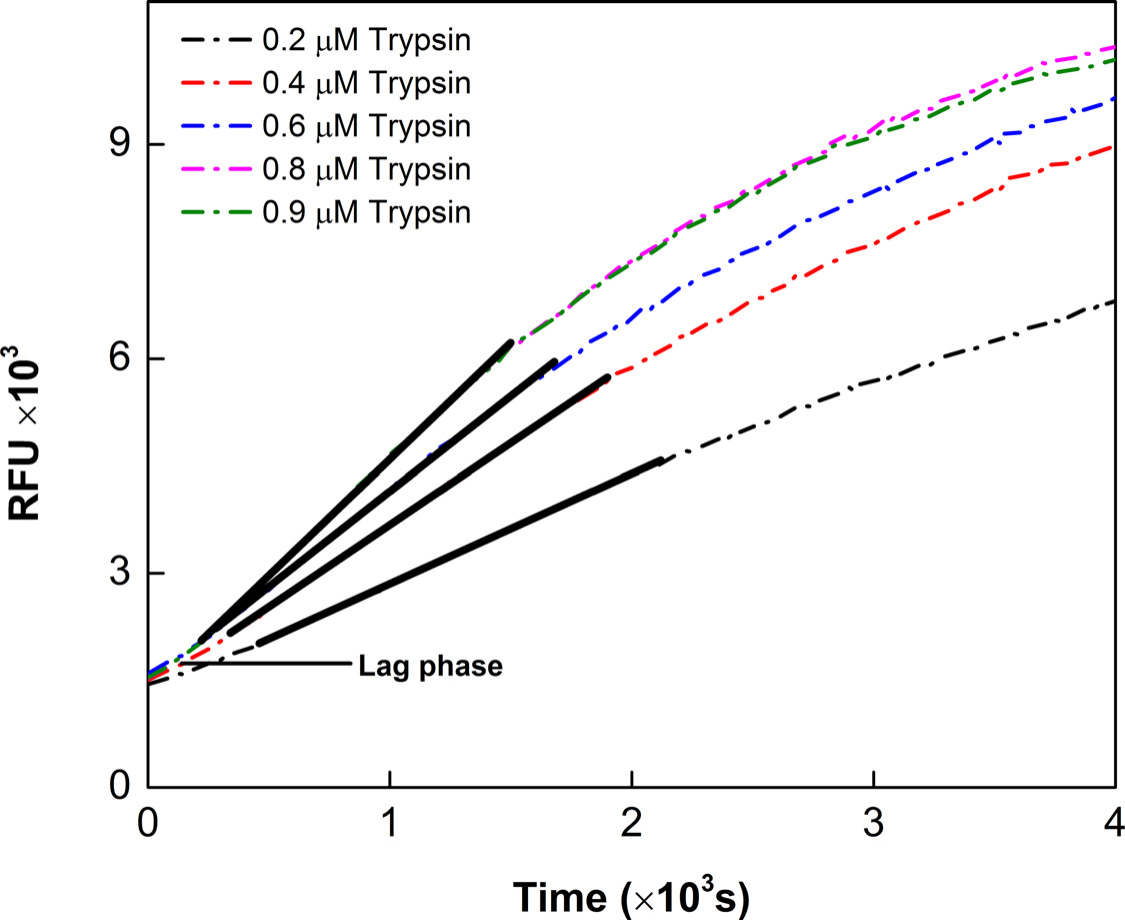
Real-time Progress Curve for SIRT5-catalyzed Desuccinylation Reaction. The time courses of the SIRT5 catalyzed reaction using the fixed concentrations of Ac-Suclys-AMC substrate and NAD^+^, and varying concentrations of trypsin as the coupling enzyme are shown. The dashed curves represent the time dependent increase in fluorescence at 460 nm (λ_ex_ = 350 nm) due to release of 7-hydroxyl-4-methylcoumarin (AMC) from the substrate. The solid lines indicate the linear regression analysis of the data after the initial lag phases (the dashed curves).

It has been noted that except for SIRT6, most of the sirtuin catalyzed reactions follow a compulsory order mechanism, involving the binding of acylated substrates followed by the binding of NAD^+^ [24–26]. To ascertain the influence of mutations on the kinetic parameters of the enzyme catalyzed reaction, we sought to perform the steady-state kinetic studies. Since the latter studies require precise determination of the initial rates of the enzyme catalysis, we employed the trypsin coupled continuous assay system [39] using acetylated-Lys (*Fluor-de-lys*^*®*^) and succinylated-Lys (Ac-Suclys-AMC) as the substrates for the deacetylation and descuccinylation reactions, respectively. While the former substrate was obtained commercially, we synthesized the succinylated substrate (Ac-Suclys-AMC) according to the procedure described by Madsen et al. [48]. In the sirtuin catalyzed assay system, trypsin cleaved the resultant lysine-coumarin conjugate, resulting in a time dependent increase in fluorescence at 460 nm (λ_ex_ = 360 nm). Fig 5 shows the representative time courses of the SIRT5 catalyzed reaction in the presence 100 μM Ac-Suclys-AMC substrate, 50 μM NAD^+^, 0.8 μM enzyme, and increasing concentrations of trypsin. Since trypsin also degrades (albeit slowly) the enzyme protein, the assay condition was optimized (with respect to the trypsin concentration) such that the initial rate of the enzyme catalyzed reaction could be reliably determined [42]. Note that at low concentration of trypsin (0.4 μM; Fig 5), the reaction proceeds with a finite lag phase. As the concentration of trypsin increases, the amplitude of the lag phase decreases and the initial steady-state rate reaches its maximum value at 0.8 μM trypsin (Fig 5, trace 4). Further increase in trypsin concentration to 0.9 μM did not affect the initial rate of the enzyme catalysis (Fig 5, trace 5) but slightly shortened the lag phase, suggesting that 0.8 μM trypsin was adequate to satisfy the coupled reaction system. In the presence of the latter concentration of trypsin, we could reliably determine the steady-state rates at time regimes significantly higher than those of the preceding lag phases as elaborated by Cleland [40]. Since our objective was to delineate the contributions of Y102 and R105 residues of SIRT5 in substrate selectivity, we opted to use acylated lysine-coumarin conjugates as the fluorogenic substrates instead of using physiologically relevant peptide substrates.

**Fig 6:**
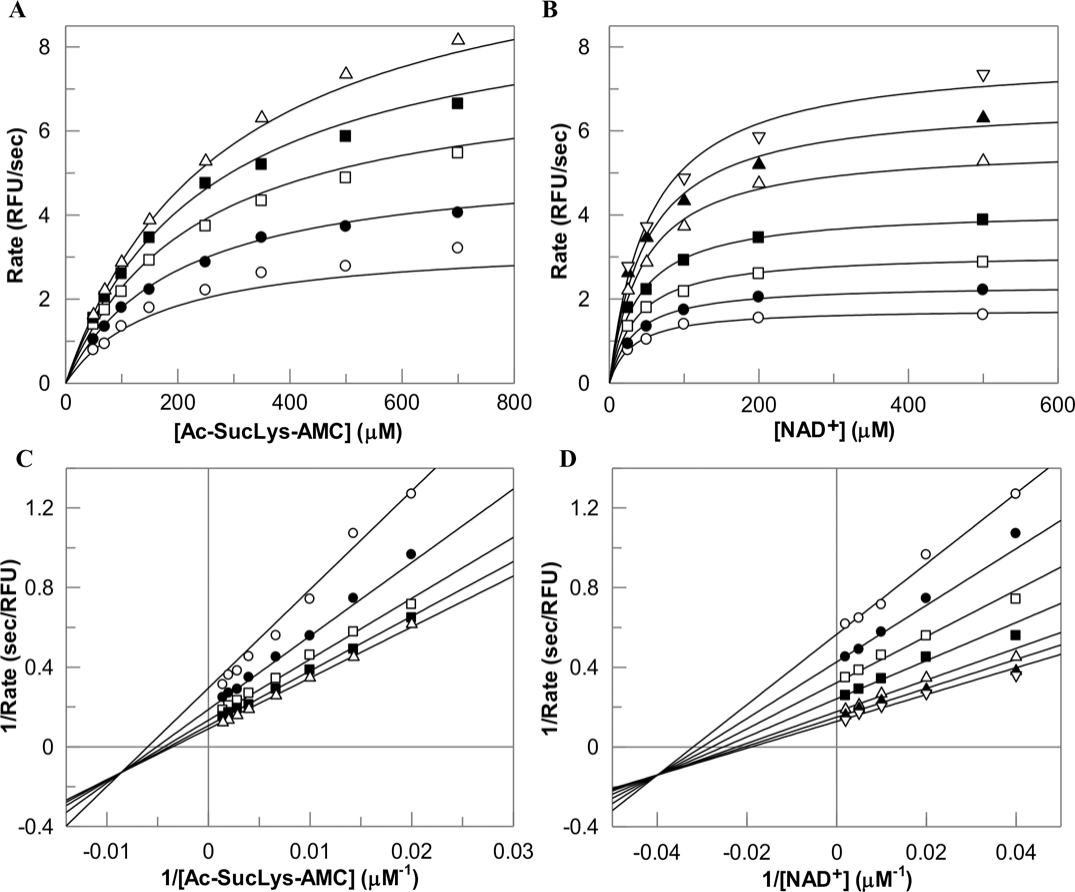
Two Substrates Reactions of SIRT5. The two substrate SIRT5 catalyzed reaction was performed under the steady-state condition with varying concentrations of Ac-SucLys-AMC substrate (25, 50, 100, 200, and 500 μM) and NAD^+^ (50, 70, 100, 150, 250, 350, 500, and 700 μM). The data were fitted to the bi-substrate ternary complex kinetic mechanism for the binding of the substrate followed by the binding of NAD^+^ by Eq 4 using Grafit software. The initial rates of SIRT5 catalysis as a function of increasing concentration of Ac-SucLys-AMC substrate at changing fixed concentrations of NAD^+^ (A), and as a function of increasing concentration of NAD^+^ at changing fixed concentrations of Ac-SucLys-AMC substrate (B) The double-reciprocal plot of 1/*v* vs 1/[NAD^+^] at increasing concentration of Ac-Suclys-AMC substrate (C), and the double-reciprocal plot of 1/*v* vs 1/[Ac-Suclys-AMC] at increasing concentration of NAD^+^ (D) are also shown.

Having optimized the reaction conditions, we determined the initial velocities of the wild-type and mutant enzyme catalyzed reactions with increasing concentrations of *Fluor-de-lys*^*®*^ or Ac-Suclys-AMC substrate and changing fixed concentrations of NAD^+^. A representative set of kinetic data for the SIRT5 catalyzed reaction as a function of Ac-Suclys-AMC substrate and NAD^+^ concentrations are shown in Fig 6. Since all sirtuins (with exception of SIRT6) catalyzed reactions conform to the bi-substrate compulsory order mechanism [24], we did not perform the product inhibition studies to substantiate the above mechanism. Instead, we analyzed the data of Fig 6 by the ternary complex kinetic mechanism (Eq 4). The solid smooth lines are the best fit of the data for the K_m,sub_ and K_m,NAD_ values as being equal to 290 ± 29 μM and 69 ± 9 μM, respectively. The k_cat_ value of the enzyme was determined to be (3.83 ± 0.38) ×10^−3^ s^-1^. We performed similar studies with Y102A, R105I, and Y102A/R015I double mutants of SIRT5 as well as with wild-type SIRT1 using both *Fluor-de-lys®* and Ac-Suclys-AMC substrates, and determined their K_m_ and k_cat_ values. These data are shown in the supporting information section figures (S1 Fig–S5 Fig), and the derived parameters are summarized in Table 2. Consistent with the previous reports [49, 50], we observed that SIRT1 possesses strong deacetylase activity with K_m,sub_ = 356 ± 56 μM and K_m,NAD_ = 388 ± 73 μM, respectively, but no detectable desuccinylase activity. On the other hand, SIRT5 showed a strong desuccinylase activity but only weak deacetylase activity, which could not be further pursued to determine its K_m_ and k_cat_ values. The data of Table 2 unravels the fact that the K_m,NAD_ for SIRT5 of desuccinylase reaction (69 μM) is about 5 fold lower than that obtained for the SIRT1 catalyzed deacetylation reaction. It should be pointed out that Madsen et al. reported the K_m,NAD_ for SIRT5 reaction using the same succinylated substrate as being equal to 150 ± 45 μM [39]. But since the above authors used only a fixed substrate concentration, the K_m,NAD_ can, at the best, be taken as its apparent value. To our further interest, we noted that Y102A mutation exhibits both deacetylase and desuccinylase activity with comparable efficiency. On the other hand, both R105I and R015/Y102A mutant enzymes were devoid of the desuccinylase activity but both these enzymes exhibited the deacetylase activity. Upon quantitative comparison, we noticed that the K_m,NAD_ for SIRT5 Y102A mutant was about 5 fold higher than that for the wild-type enzyme in case of the descuccinylation reaction, but the above discrepancy was about 20 fold in case of the deacetylase reaction. Clearly, the K_m,NAD_ is considerably higher when SIRT5 catalyzes the deacetylation versus the desuccinylation reaction. It is further noteworthy that R105I and Y102A/R105I double mutants of SIRT5 exhibit higher deacetylase activity, with k_cat_ values of (12.2 ± 1.0) × 10^−5^ s^-1^, and (2.6 ± 0.1) × 10^−5^ s^-1^, respectively, than that determined for the Y102A mutant enzyme. Although Y102A/R105I double mutant of SIRT5 mimics the active site pocket of SIRT1, the K_m,sub_ for the double mutant (388 ± 12 μM) is comparable to that obtained for SIRT1 (356 ± 56 μM), but the k_cat_ value of the former enzyme ((2.6 ± 0.1) × 10^−5^ s^-1^) was about 22 fold lower than that obtained for the latter enzyme ((5.8 ± 0.9) × 10^−4^ s^-1^). Hence, the specificity constant (k_cat_/K_m_) of Y102A/R105I double mutant SIRT5 is about 40 fold lower than that of SIRT5, suggesting that the Y102 and R105 are not exclusive determinant of the substrate specificity between SIRT1 and SIRT5. A cumulative account of these steady-state kinetic data leads to the conclusion that there is a mutual cooperation between Y102 and R105 residues in modulating the deacetylase versus desuccinylase activity of SIRT5.

**Table 2:** Steady-state Kinetic Parameters of SIRT5 Variants and SIRT1 on Acetyl and Succinyl substrates.

		K_m,sub_ (μM)	K_m,NAD_ (μM)	k_cat_ (×10^−5^ s^-1^)	k_cat_/K_m_ (M^-1^s^-1^)
**SIRT5**	**deacetylation**	**ND**^a^	**ND**^a^	**ND**^a^	**0.006**
	**desuccinylation**	**290 ± 29**	**69 ± 9**	**383.2 ± 38.3**	**13.207**
**SIRT5 Y102A**	**deacetylation**	**720 ± 144**	**1605 ± 321**	**6.6 ± 1.3**	**0.091**
	**desuccinylation**	**300 ± 54**	**321 ± 49**	**27.4 ± 4.9**	**0.409**
**SIRT5 R105I**	**deacetylation**	**1658 ± 140**	**609 ± 120**	**12.2 ± 1.0**	**0.073**
	**desuccinylation**	**ND**^a^	**ND**^a^	**ND**^a^	**0.006**
**SIRT5 Y102A R105I**	**deacetylation**	**388 ± 12**	**1194 ± 667**	**2.6 ± 0.1**	**0.065**
	**desuccinylation**	**N/A**	**N/A**	**N/A**	**0.002**
**SIRT1**	**deacetylation**	**356 ± 56**	**388 ± 73**	**57.9 ± 9.1**	**1.626**
	**desuccinylation**	**NA**^b^	**N/A**^b^	**N/A**^b^	**N/A**^b^

^a^ K_m_and k_cat_ values could not be independently determined due to the experimental limitation of using high substrate concentration; k_cat_/K_m_ was estimated from the linear first order region of the Michaelis-Menten plot at low concentrations of the substrates.

^b^ N/A: no activity was observed.

### Nicotinamide inhibition

**Fig 7:**
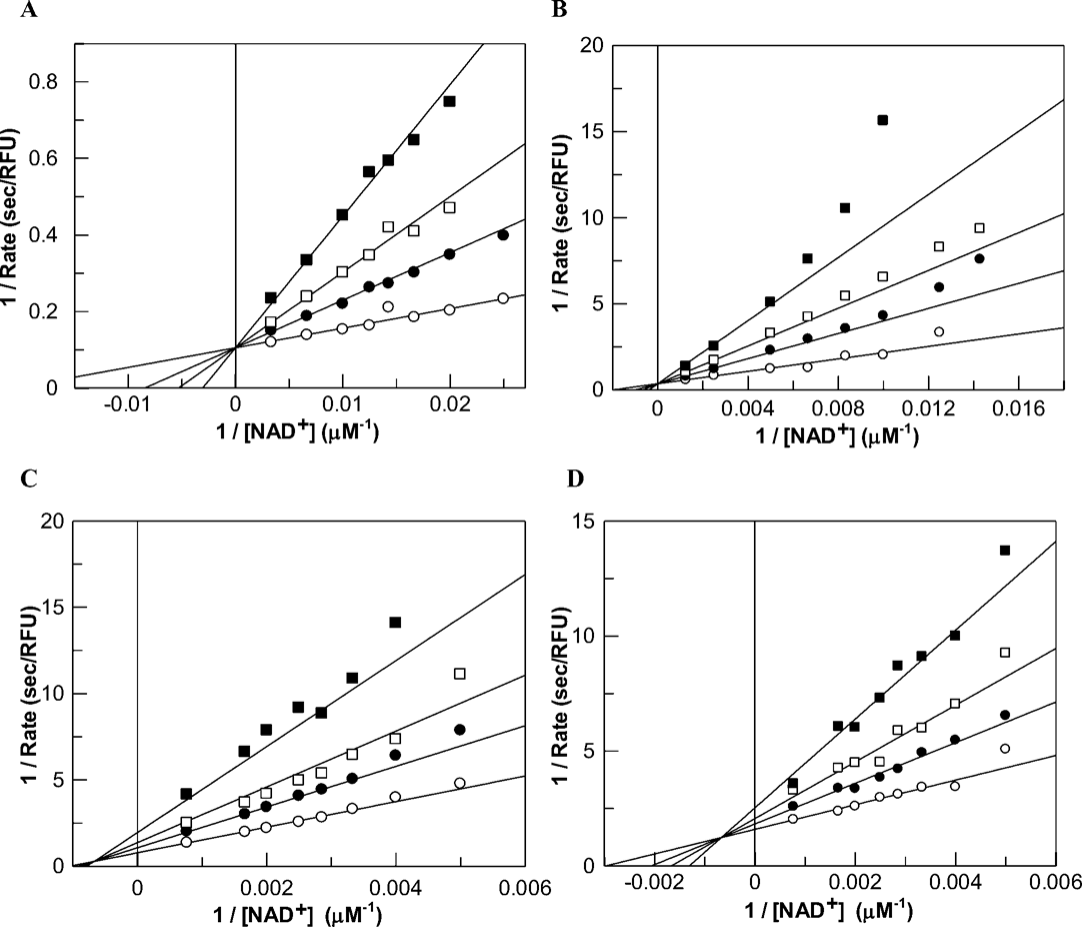
Nicotinamide Inhibition of SIRT5 Variants and SIRT1. Nicotinamide exhibits competitive inhibition against NAD^+^ during the wild-type SIRT5 and its Y102A mutant catalyzed desuccinylation reactions (A and B); mixed type of inhibition against NAD^+^ during the Y102A/R105I SIRT5 mutant and SIRT1 catalyzed deacetylation reaction (C and D). The wild-type SIRT5 catalyzed reactions (A) were performed in the presence of 100 μM Ac-SucLys-AMC and varying concentrations of NAD^+^ (from 40 to 300 μM) in the presence of 0, 25, 50 and 100 μM concentrations of nicotinamide. The Y102A mutant catalyzed reactions (B) were performed in the presence of 500 μM Ac-SucLys-AMC and varying concentrations of NAD^+^ (from 70 to 800 μM) in the presence of 0, 50, 100 and 200 μM nicotinamide. The Y102A/R105I mutant catalyzed deacetylation reactions (C) were performed in the presence of 500 μM *Fluo-de-lys*^®^ acetylated substrate, and varying concentrations of NAD^+^ concentration (from 200 to 1300 μM), in the presence of 0, 100, 200 and 400 μM nicotinamide. SIRT1 reactions (D) were performed in the presence of 100 μM *Fluo-de-lys*^®^ acetylated substrate, and varying concentrations of NAD^+^ (from 40 to 300 μM) in the presence of 0, 50, 100, and 200 μM nicotinamide. Data were fitted using DYNAFIT software and the derived K_i_ values are summarized in Table 3.

It has been proposed that the inhibition of several isoforms of sirtuins by nicotinamide is manifested via the “base-exchange” mechanism (i.e., the reversal of the step involved in the formation of nicotinamide during the enzyme catalysis; see Fig 1), and such event gives rise to noncompetitive inhibition of the enzyme [19, 20, 51]. Recently it has been proposed that with certain sirtuins (e.g., mouse SIRT2 and human SIRT3) the nicotinamide inhibition is mixed type with increasing component of the competitive inhibition [52]. To ascertain the effect of nicotinamide on human SIRT1 and SIRT5 as well as its mutants in the presence of different substrates, we performed the detailed steady-state kinetic studies via our continuous (trypsin coupled) assay system. Fig 7A shows the double reciprocal plot for the SIRT5 catalyzed desuccinylation reaction as a function of NAD^+^ concentration (in the presence of 100 μM succinylated substrate) at various fixed concentrations of nicotinamide. Although the double reciprocal plot of Fig 7A appeared to be competitive in nature, to ensure its validity, we analyzed the data by the model discrimination analysis protocol of the DYNAFIT software [43–45]. The above analysis revealed that the nicotinamide inhibition data of Fig 7A adhered to the “pure” competitive inhibition model; the data could not be reliably fitted either by non-competitive, uncompetitive, or mixed inhibition model. As will be discussed in the later section, the competitive inhibition of nicotinamide (against NAD^+^) is unlikely to be contributed by the base-exchange mechanism. When we performed similar studies for the inhibition of nicotinamide (against NAD^+^) with other mutant enzymes, we observed that Y102A also showed the competitive inhibition (Fig 7B). However, SIRT1 and SIRT5 Y102A/R105I double mutant showed the mixed type of inhibition (Fig 7C and 7D), and such inhibition could either be partly or entirely contributed by the base-exchange mechanism.

**Table 3:** Nicotinamide Inhibition against SIRT5 Variants and SIRT1.

		Inhibition Mode	Inhibition Constant(s)
**SIRT5**	**deacetylation**	**ND**	**ND**
	**desuccinylation**	**Competitive**	**(17.5 ± 1.1) μM**
**SIRT5 Y102A**	**deacetylation**	**ND**	**N/A, 36% inhibition at 800μM**
	**desuccinylation**	**Competitive**	**(49.1 ± 4.1) μM**
**SIRT5 R105I**	**deacetylation**	**ND**	**N/A, 40% inhibition at 800μM**
	**desuccinylation**	**ND**	**ND**
**SIRT5 Y102A R105I**	**deacetylation**	**Mixed**	**K**_**i**_ **= (169.3 ± 16.8) μMK**_**i’**_ **= (267.2 ± 52.4) μM**
	**desuccinylation**	**ND**	**ND**
**SIRT1**	**deacetylation**	**Mixed**	**K**_**i**_ **= (76.7 ± 12.2) μMK**_**i’**_ **= (340.5 ± 102.4) μM**
	**desuccinylation**	**ND**	**ND**

Based on their findings on different sirtuins, Sauve and Shramm concluded that while the base-exchange inhibition of nicotinamide for bacterial and yeast enzymes partially impaired their catalytic activities, it resulted in nearly 95% inhibition of the mouse enzyme [19]. The origin of such inhibitory potency of nicotinamide has been ascribed to the relative magnitude of the forward and reverse rates of formation and decay of the intermediate formed during the catalytic cycles of different sirtuins (Fig 1). It should be mentioned that we did not observe partial inhibition of nicotinamide with either SIRT1 or SIRT5 or its mutants. Fisher et al. reported that while SIRT5 deacetylase activity is insensitive to the inhibition of the enzyme by nicotinamide, the enzyme’s desuccinylase activity is highly sensitive (i.e., strongly inhibited) to nicotinamide [47]. They concluded that Arg105/succinate interaction accounted for the differential nicotinamide sensitivities of SIRT5 activities. The above conclusion appears to be corroborated by our finding of the nicotinamide inhibition with the mutant enzymes (Table 2). For example, we observed that Y102A is competitively inhibited by nicotinamide with a K_i_ value of 49.1 ± 4.1 μM in the presence of the succinylated substrate, but the above enzyme was only weakly inhibited (about 36% inhibition at 800 μM) in the presence of the acetylated substrate. Interestingly, the nicotinamide inhibition in terms of the deacetylase activity becomes more potent and conforms to the mixed inhibition mode in the case Y102A/R105I double mutant enzyme (K_i_ = 169.3 ± 16.8 μM, K_i’_ = 267.2 ± 52.4 μM). A similar mixed mode of inhibition was also observed with SIRT1 (K_i_ = 76.7 ± 12.2 μM, K_i’_ = 340.5 ± 102.4 μM) in the presence of the acetylated substrate. Apparently, both Y102 and R105 residues of SIRT5 coordinate with each other in modulating the sensitivity of nicotinamide inhibition during catalysis. Combining the aforementioned results of SIRT5 catalysis and inhibition, it is appears evident that the binding of succinylated substrate to SIRT5 favorably promotes the binding of NAD^+^ which is competitively displaced by nicotinamide. Whether the origin of the above phenomenon is kinetically or thermodynamically controlled must await further studies.

### Effect of isonicotinamide on sirtuin catalysis

**Fig 8:**
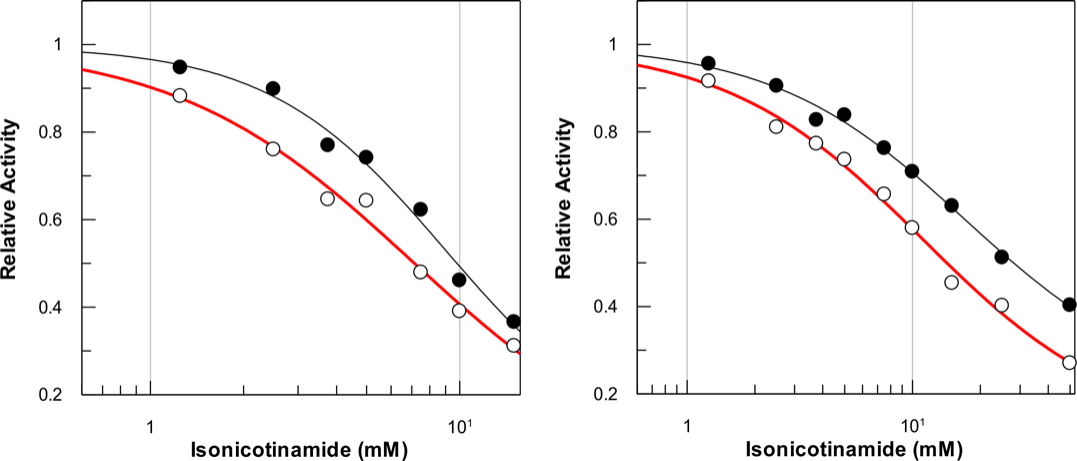
Effects of Isonicotinamide on SIRT1 and SIRT 5 Catalyzed reactions. (A) Dose-response of SIRT1 deacetylation activity in the presence of varying concentrations of isonicotinamide (from 0 to 15 mM) and NAD^+^ (50 μM and 5 mM). The solid smooth lines represent the best fit of the data for the IC_50_ values as 7.0 ± 2.9 mM (in the presence of 50 μM NAD^+^) and 8.9 ± 3.3 mM (in the presence of 5 mM NAD^+^), respectively. (B) Dose-response of SIRT5 desuccinylation activity in the presence of varying concentrations of isonicotinamide (from 0 to 15 mM) and NAD^+^ (50 μM and 5 mM). The solid smooth lines represent the best fit of the data for the IC_50_ values as 11.1 ± 2.2 mM (in the presence of 50 μM NAD^+^) and 17.1 ± 4.0 mM (in the presence of 5 mM NAD^+^), respectively.

Isonicotinamide has been reported to serve as an activator of yeast sir2 both under in vitro and in vivo conditions, and the origin of its (activating) effect has been ascribed to the alleviation of the nicotinamide assisted reverse (base-exchange) reaction [53]. To ascertain whether isonicotinamide activates SIRT1 or SIRT5, we determined the rates of the enzyme catalyzed reactions as a function of increasing concentrations of isonicotinamide. Fig 8 shows the normalized rates (represented as the relative rates) of SIRT1 catalyzed deacetylation reactions and SIRT5 catalyzed desuccinylation reactions as a function of increasing concentration of isonicotinamide in the presence of low (50 μM) and high (5 mM) concentrations of NAD^+^. The data of Fig 8 clearly shows that under neither of the above reaction conditions, the rates of the enzyme catalyzed reactions decrease upon increase in isonicotinamide concentration. This observation is contrary to that observed for the sir2 catalyzed reaction [53]. We analyzed the data of Fig 8 by Eq 5, and obtained the magnitudes of IC_50_ values as being equal to 7.0 ± 2.9 mM and 8.9 ± 3.3 mM in the presence of 50 μM and 5 mM NAD^+^, respectively, for SIRT1. The corresponding IC_50_ values for SIRT5 under above condition were found to be 11.1 ± 2.2 mM and 17.1 ± 4.0 mM, respectively. Note that although the IC_50_ values are slightly higher in the presence of higher concentration of NAD^+^, they are not significant. To further confirm that isonicotinamide does not alleviate the nicotinamide inhibition, we determined the IC_50_ values of nicotinamide for both SIRT1 (deacetylation) and SIRT5 (desuccinylation) in the absence and presence of 10 mM isonicotinamide (Table 4). The data of Table 4 suggests that there is only miniscule effect of isonicotinamide on the IC_50_ value of nicotinamide inhibition in case of both SIRT1 and SIRT5. However, since the inhibitory effect of isonicotinamide was found to be alleviated in the presence of high concentration of NAD^+^ in case of both SIRT1 and SIRT5 catalyzed reaction (Fig 8), we surmise that like nicotinamide, isonicotinamide also competes (directly) against the binding of NAD^+^ to the enzyme sites. Our results are consistent with the reports of Guan et al. revealing that isonicotinamide weakly inhibits human SIRT3 and does not alleviate the inhibition of nicotinamide [54].

**Table 4:** Isonicotinamide (iNAM) Effect on the Nicotinamide Inhibition of SIRT1 and SIRT5.

	IC_50_ of Nicotinamide	
	In the absence of iNAM	In the presence of 10 mM iNAM
**SIRT1**	**232.5 ± 52.7 μM**	**133.8 ± 27.2 μM**
**SIRT5**	**26.2 ± 2.9 μM**	**34.7 ± 3.4 μM**

### Roles of Arg105 and Tyr102 of SIRT5 in modulating the inhibitory potency of EX527

**Fig 9:**
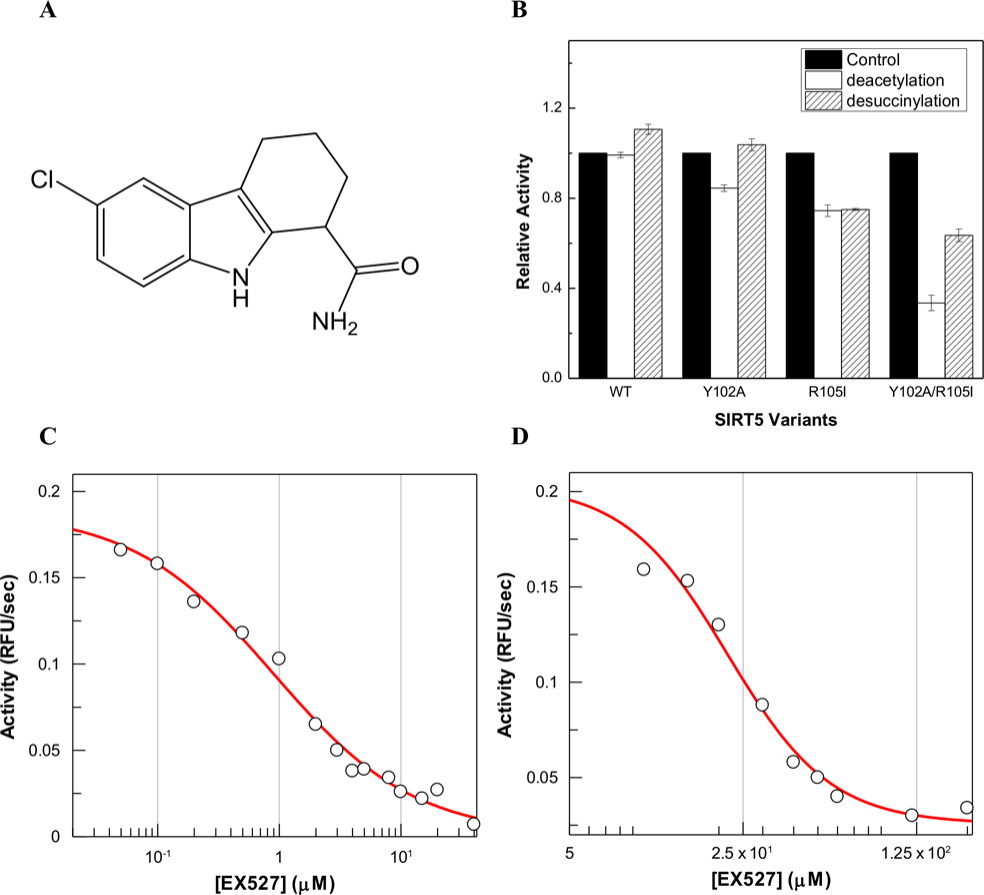
Structure of EX527 and its Effects on SIRT5 Variant Catalyzed Reactions. (A) Structure of EX527. (B) Relative activities of SIRT5 variants in the absence and presence of 50 μM EX527. (C) Dose-response of SIRT1 deacetylase activity in the presence of increasing concentration of EX527 (from 0 to 40 μM). (D) Dose-response of SIRT5 Y102A/R105I deacetylase activity in the presence of increasing concentration of EX527 (from 0 to 200 μM). For (C) and (D), the solid smooth lines represent the best fit of the data for the IC_50_ of EX527 against SIRT1 and SIRT5 Y102A/R105I as being equal to 0.9 ± 0.2 μM and 21.7 ± 1.0 μM, respectively.

EX527 (6-chloro-2,3,4,9-tetrahydro-1H-carbazole-1-carboxamide; Fig 9A) is one of few compounds that possess both high inhibitory potency as well as isoform selectivity against sirtuins [55]. EX527 inhibits SIRT1 but shows no effect on either deacetylase or desuccinylase activities of SIRT5 [56]. It has been suggested that the insensitivity of SIRT5 against EX527 is due to the unique Arg105 residue within the active site pocket of the enzyme. However, to the best of our knowledge, there has been no experimental data to support or refute the above hypothesis. To probe the contributions of R105 and/or Y102 of SIRT5 in modulating the inhibitory potency of EX527, we measured the rates of Y012A, R105I, and Y102A/R015I mutant catalyzed reactions as a function of increasing concentrations of EX527. Fig 9B shows the relative activities of SIRT5 variants in the absence and presence of 50 μM EX527. As reported previously, we observed that wild-type SIRT5 was not inhibited by EX527 [56]. On the other hand, EX527 showed about 14% inhibition against Y102A mutant of SIRT5 with *fluor-de-lys* substrate, but showed no inhibition with succinylated substrate. We further observed that R105I mutant was found to be inhibited by about 22% by 50 μM EX527 in terms of both deacetylase and desuccinylase activities. On the other hand, SIRT5 double mutant Y102A/R105I exhibited EX527 sensitivity comparable to that of human SIRT3. The IC_50_ value for the inhibition of the double mutant enzyme (21.7 ± 1.0 μM) was found to be similar to that reported (22.4 ± 2.7 μM) for the inhibition of SIRT3. However, the inhibitory potency of EX527 for SIRT1 (IC_50_ = 0.9 ± 0.1 μM) is still significantly higher than that accountable for the exclusive contributions of Y102 and/or R105 in SIRT5. These data suggest that both Tyr102 and Arg105 mutually contribute to the sensitivity of inhibition by EX527 in SIRT1 versus SIRT5 enzymes during deacetylase reaction.

**Fig 10:**
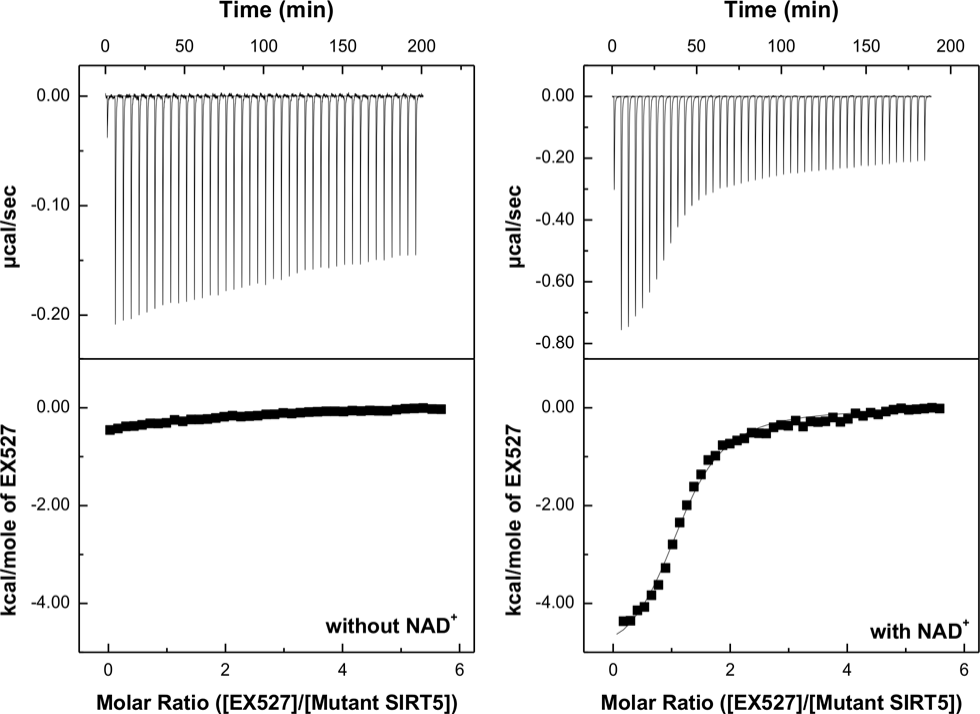
ITC profile for the binding of EX527 to SIRT5 Y102A/R105I in the absence (left) and presence of 10 mM NAD^+^ (right). The top panels show the raw calorimetric data generated by titration of 20 μM SIRT5 Y102A/R105I by 45 injections (5 μl each) of 500 μM EX527. The area under each peak was integrated and plotted against the molar ratio of EX527 to SIRT5 Y102A/R105I. The solid lines (right panel) represent the best fit of the experimental data for the single binding site model, yielding the magnitudes of n, ΔH° and K_a_ as being equal to 1.2, −5.3 ± 0.1 kcal/mol and (2.5 ± 0.2) × 10^5^ M^-1^, respectively.

Given a reasonably tighter binding affinity of EX527 to SIRT5 Y102A/R105I double mutant (as evident by much lower IC_50_ value), we purported to determine their thermodynamic parameters by performing isothermal titration calorimetry (ITC) studies. Fig 10 shows the ITC profile for the titration of SIRT5 Y102A/R105I double mutant by increasing 45 aliquots of 1 mM EX527 in the absence and presence of 10 mM NAD^+^ in 50 mM HEPES buffer, pH 7.5, containing 100 mM NaCl, 10% glycerol and 1 mM TCEP at 25°C. The raw calorimetric data for the interaction of EX527 to SIRT5 Y102A/R105I double mutant and their binding isotherms are shown in the top and the bottom panels of Fig 10, respectively. The left panel of Fig 10 shows that EX527 binds weakly to SIRT5 Y102A/R105I mutant in the absence of NAD^+^. However, in the presence of 10 mM NAD^+^ (Fig 10, right panel), EX527 produces higher magnitude of the heat signal, suggesting tighter binding affinity of the inhibitor to the double mutant enzyme. The data of Fig 10 (left) was analyzed for the single site binding model, yielding the magnitudes of n, ΔH° and K_a_ as being equal to 1.2, −5.3 ± 0.1 kcal/mol and (2.5 ± 0.2) × 10^5^ M^-1^, respectively. The latter value translates to the ΔG° of binding as being equal to −7.3 ± 0.1 kcal/mol. Given the ΔH° and ΔG° values, the TΔS° was calculated to be 2.03 kcal/mol. In view of these thermodynamic parameters, it is evident that the binding of EX527 to Y102A/R105 double mutant of SIRT5 is dominated by the enthalpic contribution to the overall binding free energy. When we performed the above experiment for the binding of EX527 to the wild type SIRT5 under an identical condition, only miniscule heat signals were generated either in the absence or the presence of NAD^+^ (S6 Fig). Hence, Y102 and R105 preclude the binding of EX527 to wild-type SIRT5.

## Discussion

We provide, for the first time, a comprehensive investigation on the role of two crucial amino acids (viz., Y102 and R105) of SIRT5 which alter the substrate specificity of the enzyme in catalyzing the desuccinylation as opposed to the deacetylation (catalyzed by other sirtuin isozymes) reaction. Since the above amino acids are substituted by Ala and Ile in SIRT1, we purported to probe the independent and cumulative contributions of Y102 and R105 residues of SIRT5 in defining the substrate specificity as well as other structural-functional features of the enzyme. A comparative account of our experimental data presented in the previous section leads to the following conclusions: (1) Unlike R105I and Y102A/R105I mutant enzymes (which predominantly catalyze the deacetylase reaction), Y102A mutant enzyme catalyzes both deacetylation and descuccinylation reactions with comparable efficiency. (2) Contrary to the evidence derived from the structural data [14], our kinetic data does not support the role of Y102 in strengthening the binding of negatively charged succinylated substrate to SIRT5; instead the above residue favors the binding of NAD^+^ both during the deacetylation and descuccinylation reactions. (3) In cases where we could reliably determine the mode of nicotinamide inhibition, only the deacetylation reaction (catalyzed by SIRT1 and SIRT5 Y102A/R015I mutant) showed the mixed inhibition of nicotinamide against NAD^+^; the descuccinylation reaction (catalyzed by wild-type SIRT5 and its SIRT5 Y102A mutant) unequivocally showed the competitive inhibition of nicotinamide against NAD^+^, suggesting that the “base-exchange” mechanism is either nonexistent or negligible in the latter cases. (5) Isonicotinamide served as an inhibitor rather than the activator for both SIRT1 and SIRT5 catalyzed reactions. (6) Except for SIRT1, the CD spectra of wild-type SIRT5 and its mutants showed only subtle differences. (7) Whereas the temperature dependent changes in the CD spectra of the wild-type SIRT1 and SIRT5, and SIRT5 Y102A mutant conformed to a single melting transition profile, the R105 mutants of SIRT5 (R105I and Y102A/R105I) were consistent with two melting transitions with comparable amplitudes but markedly different T_m_ values. (8) The inhibitory potency of a SIRT1 specific inhibitor, EX527, increases with increase in the deacetylation activity of SIRT5 mutants.

It has been widely accepted that except for SIRT6 all sirtuin-catalyzed reactions follow a compulsory order mechanism in which the acylated peptide binds first followed by the binding of NAD^+^ to generate the enzyme-substrate-NAD^+^ ternary complex, which undergoes the chemical transformation reaction to produce the reaction products [24–26]. Since all sirtuins share a highly conserved catalytic core, it is not surprising that they conform to the same bi-substrate ternary complex mechanism. Hence, we did not attempt to differentiate among alternative bi-substrate reaction mechanisms with wild-type SIRT1 and SIRT5 and its mutants by performing product inhibition studies. Instead, we analyzed all our kinetic data (collected in a matrix format with varying concentrations of the substrate(s) and NAD^+^) by two-substrate ternary complex mechanism to derive the kinetic parameters of the enzymes. The data of Table 2 clearly reveals that for structurally similar fluorogenic substrates, the specificity constant (k_cat_/K_m_) for the SIRT5 catalyzed desuccinylation reaction is about one order of magnitude higher than that for the SIRT1 catalyzed deacetylation reaction, and none of the SIRT5 mutants (either catalyzing the descuccinylation or deacetylation reaction) yield the specificity constants similar to or higher than the wild-type enzymes. Hence, although Y102A and/or R105I mutations in SIRT5 promote the deacetylase activity, the catalytic machinery of the mutant enzymes is not as efficient as that of SIRT1. Clearly, the substrate specificity of SIRT1 and SIRT5 is not manifested exclusively by the (above noted) “two” residues. Furthermore, K_m_ for NAD^+^ during the descuccinylation reaction (given by the wild-type SIRT5 and Y102A mutant) is lower than that for the deacetylation (given by SIRT1 and Y102A and Y102A/R105I mutants of SIRT5) reaction. This difference is presumably due to the electrostatic interaction between R105 and the phosphate moieties of NAD^+^ during the descuccinylation reaction.

Since the kinetic mechanism of sirtuin catalysis involves the release of the nicotinamide moiety from NAD^+^ upon formation of the *O*-acylimidate intermediate (Fig 1), the mechanistic as well as the physiological role of nicotinamide has been frequently discussed in the literature [19, 20, 23, 47, 53, 54, 57]. Schramm and Denu’s groups have elegantly demonstrated that the nicotinamide inhibition of Sir2 is caused by the reversal of the acyl transfer step (i.e., the conversion of enzyme-substrate-NAD^+^ ternary complex to enzyme-acylimidate-nicotinamide complex; referred to as the “base-exchange” mechanism), and such inhibition exhibits the non-competitive or mixed type of behavior [19, 20]. Furthermore, it has been observed that the inhibition of the enzyme by nicotinamide (via the base-exchange mechanism) does not result in 100% loss in the enzyme activity, and the enzyme is often activated in the presence of isonicotinamide (a non-reactive analog of nicotinamide). Subsequent studies on different isoforms of sirtuins show markedly different inhibition profiles, which question the ubiquity of the nicotinamide inhibition exclusively via the base-exchange mechanism [47, 54]. This is particularly so when the nicotinamide inhibition (against NAD^+^) is purely competitive in nature. Our model discrimination analysis for the wild-type SIRT5 and its Y012A mutant catalyzed reactions unambiguously demonstrate that the nicotinamide inhibition (against NAD^+^) is purely “competitive” in nature, and the enzyme is nearly 100% inhibited in the presence of saturating concentration of the inhibitor. We note that Guan et al. argued, based on the kinetic and modeling grounds, that “apparent competitive” inhibition of nicotinamide (against NAD^+^) in human SIRT3 catalyzed reaction may also involve the contribution of the base-exchange mechanism [54]. However, it is non-intuitive as to how a “pure” competitive inhibitor, which inhibits the enzyme activity by nearly 100%, would reverse the deacylation step by exclusively interacting with the enzyme-acylimidate complex but not with the enzyme-substrate complex. The possibility of nicotinamide competitively displacing NAD^+^ from the enzyme-substrate-NAD^+^ complex is further supported by our isothermal titration calorimetric data (S5 Fig) for the binding of nicotinamide to SIRT5-substrate complex. The ITC data shows that the K_d_ value (41 μM) for the binding of nicotinamide to the enzyme-substrate complex is comparable to the K_m_ value of NAD^+^ (69 μM) during the wild-type SIRT5 catalyzed descuccinylation reaction. Unlike the descuccinylation reaction, our nicotinamide inhibition data for the deacetylation reaction shows the mixed type of inhibition as observed with Sir2 enzymes in which the base-exchange mechanism has been unambiguously demonstrated [20].

The fact that SIRT1 selective inhibitor, EX527, inhibits the SIRT3 catalyzed reaction but neither binds to wild-type SIRT5 nor inhibits its catalysis [56] is consistent with our finding that Y102 and R105 are responsible for precluding its avidity to the latter enzyme site. Since the magnitude of inhibition of SIRT5 R05I mutant by EX527 is higher than that determined with SIRT5 Y102A mutant (Fig 9B), it appears logical to conclude that R105 in SIRT5 is a major contributor of resisting the inhibition. But this simplistic justification falls short in explaining a marked increase in the inhibitory potency of EX527 for Y012A/R105I double mutant enzyme. Evidently, Y102 and R105 coordinate with each other in controlling the accessibility (consequently inhibition) of the inhibitor to the enzyme. Interestingly, the inhibition constant of EX527 for Y102A/R105I mutant enzyme (21.7 ± 1.0 μM) is comparable to that obtained with the wild-type SIRT3, albeit it is still considerably higher than that obtained with SIRT1. Furthermore, our ITC data for the binding of EX527 to Y102A/R105I double mutant enzyme reveals that the overall binding free energy is dominated by enthalpic rather than entropic contribution. We conjecture that the lack of inhibition of wild-type SIRT5 by EX527 is contributed both by the steric hindrance (imposed by Y102 and R105 residues) as well as by relatively higher level of solvation of the enzyme’s active site pocket which is not conducive for binding of the predominantly hydrophobic molecules like EX527.

In view of the above discussion, it may imply that the differential substrate specificity as well as the inhibitory features of nicotinamide and EX527 between SIRT1 and SIRT5 are qualitatively mediated by the two amino acid residues within the active site pockets of the above enzymes. Whether such amino acid residues just alter the microenvironments of the enzyme’s active site pockets or they modulate the global conformational states of the enzymes becomes evident by examining the secondary structural features of the wild-type SIRT1 and SIRT5 and its mutants. Although the difference in the CD spectra between the wild-type SIRT1 and SIRT5 can be ascribed to the prevalence of the higher degree of random coil structure (due to extended N and C terminal residues) in case of the former enzyme, the difference in the CD spectral features among SIRT5 mutant enzymes clearly indicate to the subtle, albeit discernible, difference in the protein structures. The latter feature becomes more pronounced upon examining the temperature dependent changes in the secondary structures of the wild-type and mutant enzymes. It is explicitly evident that while wild-type SIRT1 and SIRT5 as well as Y102A mutant show the single step transition profile during the temperature dependent changes in the secondary structures of the protein, both Arg mutants (viz., R105I and Y102A/R015I) conform to the double step transitions with significantly different T_m_ values. It is noteworthy that Y102A mutation decreases the T_m_ value of the wild-type SIRT5 by about 11 degree, suggesting that the above mutation has destabilizing effect on the protein structure. While the T_m_ of the first phases of R105I and Y102A/R105I is similar to the T_m_ values of wild type and Y102A mutant enzymes, respectively, the T_m_ value of the second phases of the Arg mutants are both above 70°C.

The question arises why R105 mutations in SIRT5 cause two phase thermal transitions. We hypothesize that its origin may lie in two plausible scenarios: (1) the R105 mutation results in the reorganization of the protein domains such that they undergo independent thermal unfolding/transitions at two temperatures, (2) the R105 mutation creates two slowly interconvertible protein conformations which unfold differently. However, none of the above scenarios is consistent with the CD spectral data of the unfolded enzymes. The CD spectral data of wild-type SIRT5 as well as Y102A mutant enzymes show higher contribution of the random coil structure. But this is not the case with the R105I mutant enzyme. The CD spectra after heating of R105I and Y102A/R105I mutant enzymes (followed by cooling at 25°C) show dominant α helical and β sheet structures, but rarely any contribution of the random coil structure (as observed with the heated samples of wild-type SIRT5 and its Y102 mutant; Fig 4F). Hence, we are tempted to speculate that R105 mutant enzyme (cooperatively) acquires a more stable protein conformation (which melts at a much higher temperature) while undergoing unfolding of the native conformational state of the enzyme. This is presumably the reason why melting transition profile of the double mutant enzyme is not reliably fitted by the (independent) double transition Boltzmann equation (Eq 3). Irrespectively, the denaturation does not preserve the active site pocket of the double mutant enzyme to facilitate catalysis.

In summary, our detailed comparative studies between SIRT1 and SIRT5 sheds light on the selectivity of the enzymes for different substrates and their distinct modes of inhibition by nicotinamide and EX527 inhibitors. The experimental data clearly suggest a marked cooperation/coordination between the Y102 and R105 residues in modulating the above features, and the latter appears to be facilitated by changes in the protein structure. Whether or not such structural differences are discernible via X-ray crystallographic analysis must await further studies.

## Supporting Information

S1 Fig Two substrate deacetylation reactions of SIRT5 Y102A mutant. The two substrate SIRT5 Y102A mutant enzyme catalyzed reactions were performed under the steady-state conditions with varying concentrations of NAD^+^ (400, 600, 800, 1000, 1500 and 4000 μM) and *Fluor-de-lys*^*®*^ substrate (100, 200, 400, 600, 800 and 1200 μM). The hyperbolic dependence of the enzymatic reaction as a function of *Fluor-de-lys*^®^ concentration at changing fixed concentration of NAD^+^ (A), and that as a function of NAD^+^ concentration at changing fixed concentration of *Fluor-de-lys*^®^ substrate (B) are shown. The double-reciprocal plots of the data of panels (A) and (B) are shown in panels (C) and (D), respectively. The solid smooth lines are the best fit of the data for the sequential two-substrate mechanism using the Grafit software.
(TIF)

S2 Fig Two Substrate Desuccinylation Reactions of SIRT5 Y102A mutant. The two substrate SIRT5 Y102A mutant enzyme catalyzed reactions were performed under the steady-state conditions with varying concentrations of NAD^+^ (400, 600, 800, 1000, 1500 and 4000 μM) and Ac-SucLys-AMC substrate (100, 200, 400, 600, 800 and 1000 μM). The hyperbolic dependence of the enzymatic reaction as a function of Ac-SucLys-AMC concentration at changing fixed concentration of NAD^+^ (A), and that as a function of NAD^+^ concentration at changing fixed concentration of the substrate (B) are shown. The double-reciprocal plots of the data of panels (A) and (B) are shown in panels (C) and (D), respectively. The solid smooth lines are the best fit of the data for the sequential two-substrate mechanism using the Grafit software.
(TIF)

S3 Fig Two Substrate Deacetylation Reaction of SIRT5 R105I mutant. The two substrate SIRT5 R105I mutant enzyme catalyzed reactions were performed under the steady-state conditions with varying concentrations of NAD^+^ (400, 600, 800, 1000, 1500 and 4000 μM) and *Fluor-de-lys*^®^ substrate (100, 200, 400, 600, 800 and 1200 μM). The hyperbolic dependence of the enzymatic reaction as a function *Fluor-de-lys*^®^ concentration at changing fixed concentration of NAD^+^ (A), and that as a function of NAD^+^ concentration at changing fixed concentration of the substrate (B) are shown. The double-reciprocal plots of the data of panels (A) and (B) are shown in panels (C) and (D), respectively. The solid smooth lines are the best fit of the data for the sequential two-substrate mechanism using the Grafit software.
(TIF)

S4 Fig Two Substrate Deacetylation Reactions of SIRT5 Y102A/R105I double mutant. The two substrate SIRT5 Y102A/R105I double mutant enzyme catalyzed reactions were performed under the steady-state conditions with varying concentrations of NAD^+^ (400, 600, 800, 1000, 1500 and 4000 μM) and *Fluor-de-lys*^®^ substrate (100, 200, 400, 600, 800 and 1000 μM). The hyperbolic dependence of the enzymatic reaction as a function of *Fluor-de-lys*^®^ concentration at changing fixed concentration of NAD^+^ (A), and that as a function of NAD^+^ concentration at changing fixed concentration of the substrate (B) are shown. The double-reciprocal plots of the data of panels (A) and (B) are shown in panels (C) and (D), respectively. The solid smooth lines are the best fit of the data for the sequential two-substrate mechanism using the Grafit software.
(TIF)

S5 Fig Two Substrates Deacetylation Reactions of SIRT1. The two substrate SIRT1 catalyzed reactions were performed under the steady-state condition with varying concentrations of NAD^+^ (400, 600, 800, 1000, 1500 and 4000 μM) and *Fluor-de-lys*^®^ substrate (100, 200, 400, 600, 800 and 1000 μM). The hyperbolic dependence of the enzymatic reaction as a function of *Fluor-de-lys*^®^ concentration at changing fixed concentration of NAD^+^ (A), and that as a function of NAD^+^ concentration at changing fixed concentration of the substrate (B) are shown. The double-reciprocal plots of the data of panels (A) and (B) are shown in panels (C) and (D), respectively. The solid smooth lines are the best fit of the data for the sequential two-substrate mechanism using the Grafit software.
(TIF)

S6 Fig ITC profile for the Binding of Nicotinamide to SIRT5-substrate Complex. The top panels show the heat signals generated upon titration of 20 μM SIRT5 by 45 injections (5 μl each) of 2 mM nicotinamide in the presence of 1 mM Ac-Suclys-AMC. The area under each peak was integrated and plotted against the molar ratio of nicotinamide to SIRT5. The solid line represent the best fit of the experimental data for the single site binding model of nicotinamide to the enzyme-substrate complex, yielding the magnitudes of K_a_ and ΔH° as being equal to −(1.3 ± 0.1) kcal/mol and (2.5 ± 0.3) × 10^4^ M^-1^, respectively.
(TIF)

S7 Fig **ITC Profile for the Binding of EX527 to SIRT5 in the Absence (A) and Presence of 10 mM NAD**^**+**^ **(B).** The top panels show the heat signals generated upon titration of 20 μM SIRT5 Y102A/R105I double mutant by 45 injections (5 μl each) of 500 μM EX527. The area under each peak was integrated and plotted against the molar ratio of EX527 to SIRT5 Y102A/R105I. Due to miniscule change in the heat signals, the ITC data could not be reliably analyzed by any thermodynamic model.
(TIF)
