# Supplementary figures and images for: Role of the Substrate Specificity-Defining Residues of Human SIRT5 in Modulating the Structural Stability and Inhibitory Features of the Enzyme
Source: PLoS One. 2016 Mar 29;11(3):e0152467. doi: 10.1371/journal.pone.0152467 (PMC4811591; doi:10.1371/journal.pone.0152467)

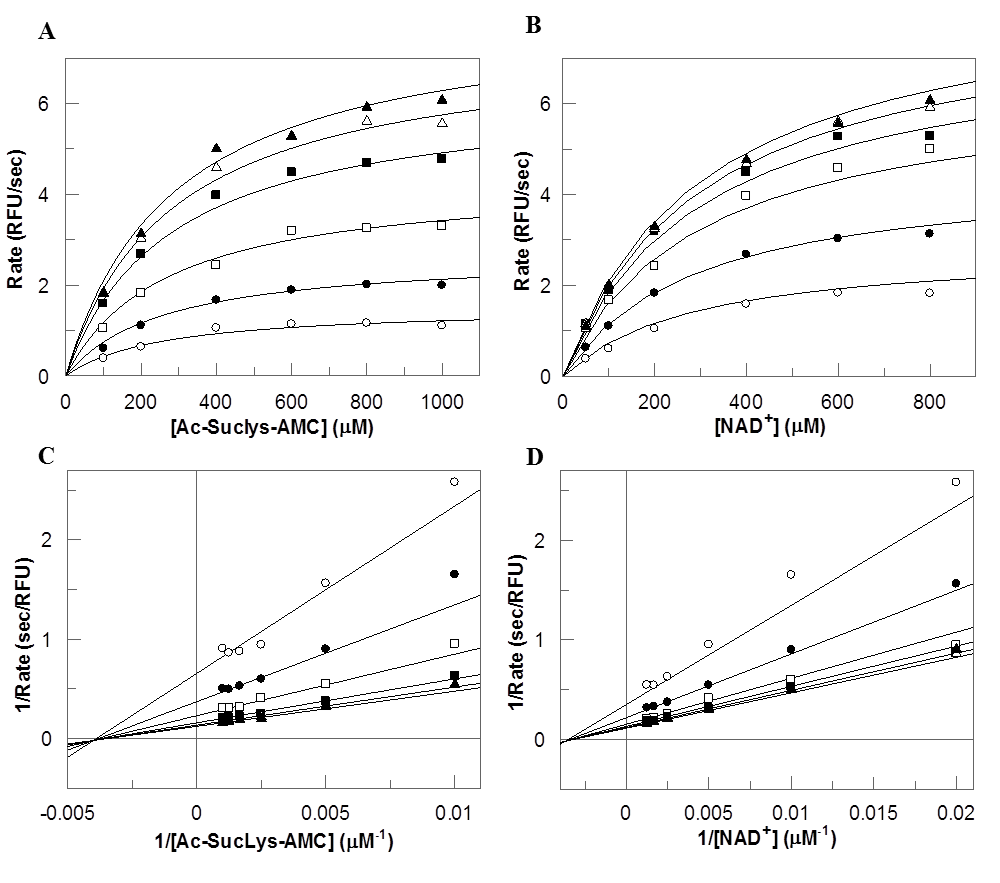

Supplement: S1 Fig — The two substrate SIRT5 Y102A mutant enzyme catalyzed reactions were performed under the steady-state conditions with varying concentrations of NAD+ (400, 600, 800, 1000, 1500 and 4000 μM) and Fluor-de-lys® substrate (100, 200, 400, 600, 800 and 1200 μM). The hyperbolic dependence of the enzymatic reaction as a function of Fluor-de-lys® concentration at changing fixed concentration of NAD+ (A), and that as a function of NAD+ concentration at changing fixed concentration of Fluor-de-lys® substrate (B) are shown. The double-reciprocal plots of the data of panels (A) and (B) are shown in panels (C) and (D), respectively. The solid smooth lines are the best fit of the data for the sequential two-substrate mechanism using the Grafit software. (TIF) [file pone.0152467.s001.tif]

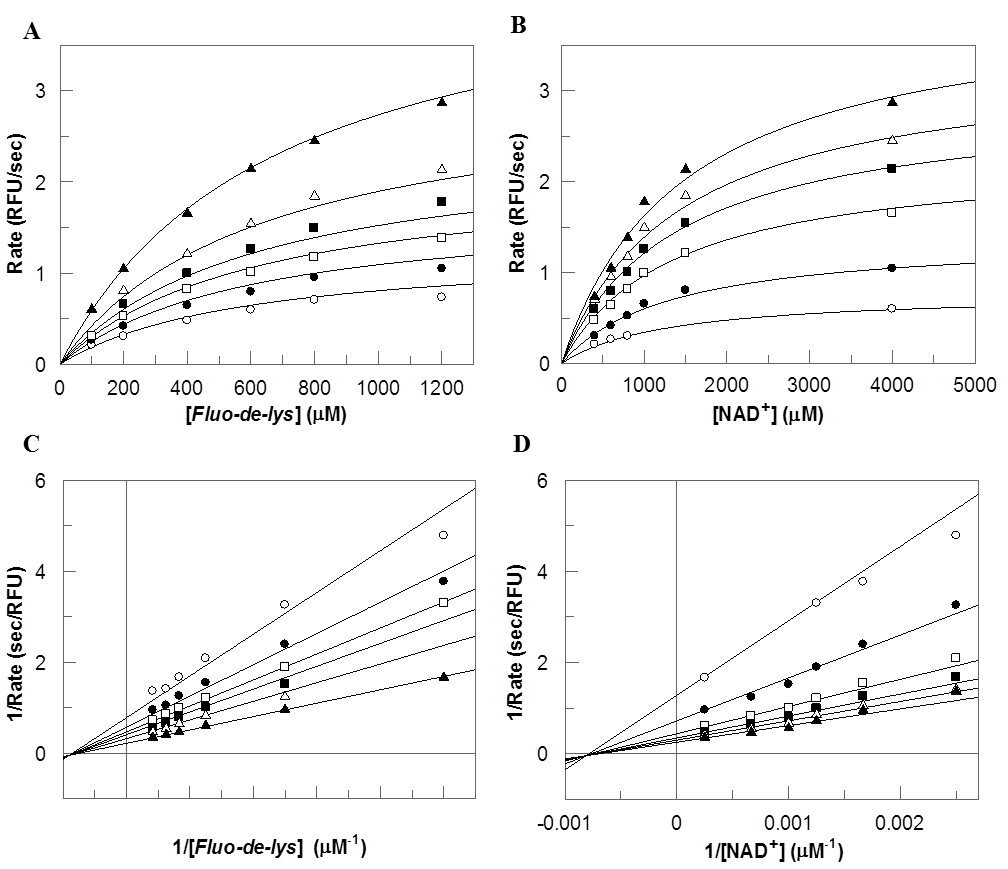

Supplement: S2 Fig — The two substrate SIRT5 Y102A mutant enzyme catalyzed reactions were performed under the steady-state conditions with varying concentrations of NAD+ (400, 600, 800, 1000, 1500 and 4000 μM) and Ac-SucLys-AMC substrate (100, 200, 400, 600, 800 and 1000 μM). The hyperbolic dependence of the enzymatic reaction as a function of Ac-SucLys-AMC concentration at changing fixed concentration of NAD+ (A), and that as a function of NAD+ concentration at changing fixed concentration of the substrate (B) are shown. The double-reciprocal plots of the data of panels (A) and (B) are shown in panels (C) and (D), respectively. The solid smooth lines are the best fit of the data for the sequential two-substrate mechanism using the Grafit software. (TIF) [file pone.0152467.s002.tif]

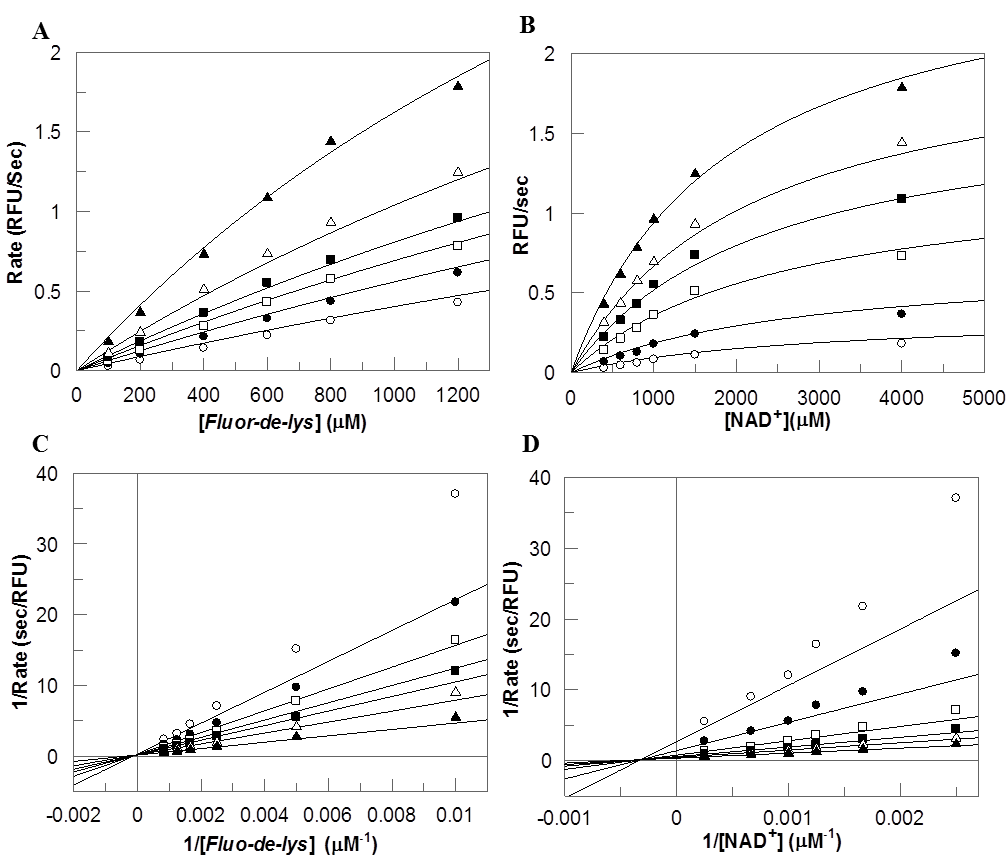

Supplement: S3 Fig — The two substrate SIRT5 R105I mutant enzyme catalyzed reactions were performed under the steady-state conditions with varying concentrations of NAD+ (400, 600, 800, 1000, 1500 and 4000 μM) and Fluor-de-lys® substrate (100, 200, 400, 600, 800 and 1200 μM). The hyperbolic dependence of the enzymatic reaction as a function Fluor-de-lys® concentration at changing fixed concentration of NAD+ (A), and that as a function of NAD+ concentration at changing fixed concentration of the substrate (B) are shown. The double-reciprocal plots of the data of panels (A) and (B) are shown in panels (C) and (D), respectively. The solid smooth lines are the best fit of the data for the sequential two-substrate mechanism using the Grafit software. (TIF) [file pone.0152467.s003.tif]

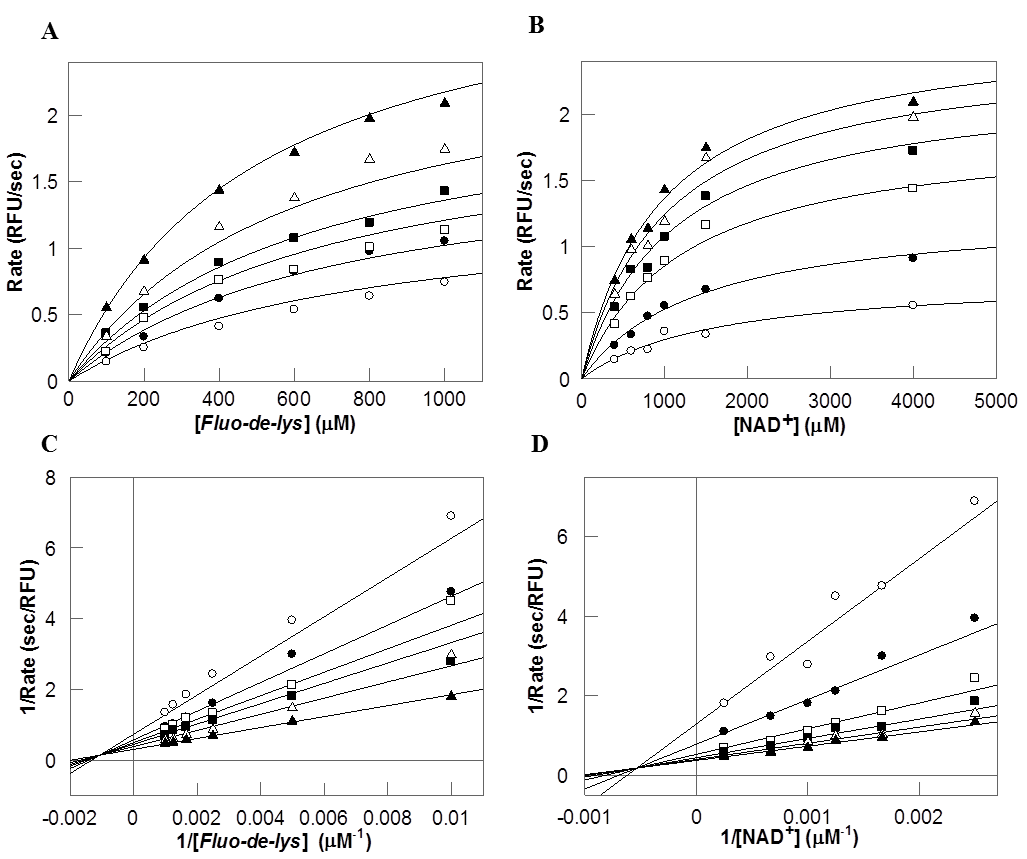

Supplement: S4 Fig — The two substrate SIRT5 Y102A/R105I double mutant enzyme catalyzed reactions were performed under the steady-state conditions with varying concentrations of NAD+ (400, 600, 800, 1000, 1500 and 4000 μM) and Fluor-de-lys® substrate (100, 200, 400, 600, 800 and 1000 μM). The hyperbolic dependence of the enzymatic reaction as a function of Fluor-de-lys® concentration at changing fixed concentration of NAD+ (A), and that as a function of NAD+ concentration at changing fixed concentration of the substrate (B) are shown. The double-reciprocal plots of the data of panels (A) and (B) are shown in panels (C) and (D), respectively. The solid smooth lines are the best fit of the data for the sequential two-substrate mechanism using the Grafit software. (TIF) [file pone.0152467.s004.tif]

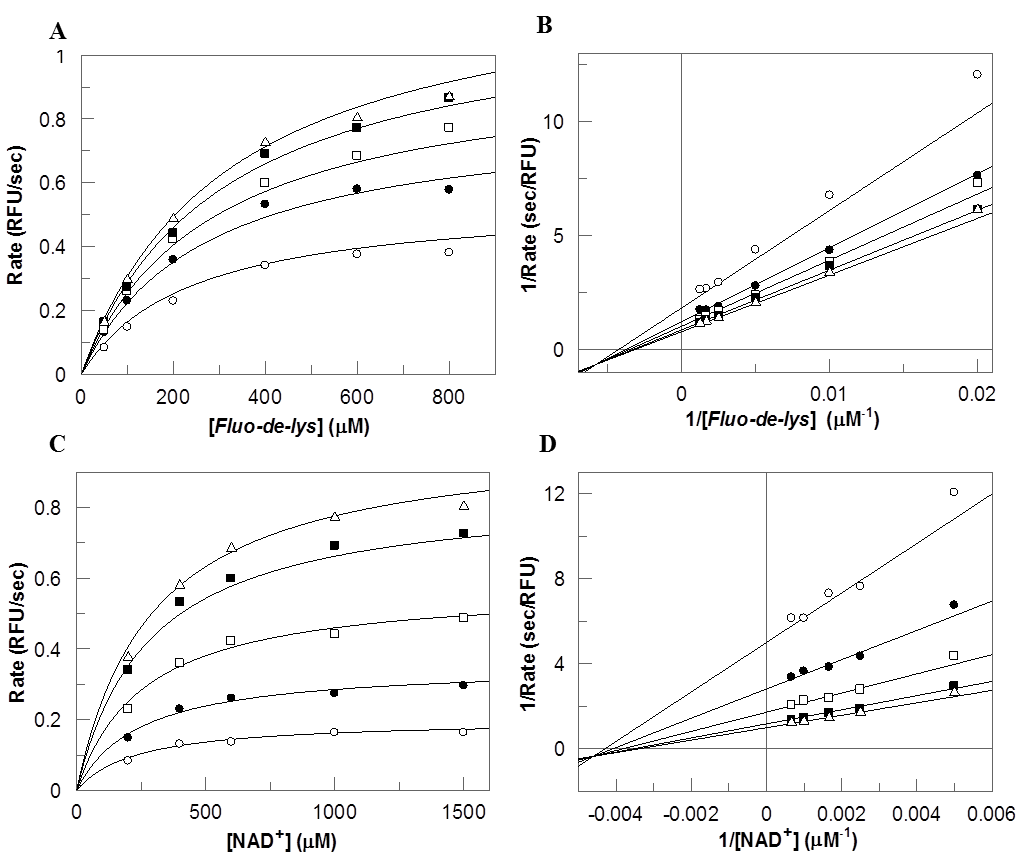

Supplement: S5 Fig — The two substrate SIRT1 catalyzed reactions were performed under the steady-state condition with varying concentrations of NAD+ (400, 600, 800, 1000, 1500 and 4000 μM) and Fluor-de-lys® substrate (100, 200, 400, 600, 800 and 1000 μM). The hyperbolic dependence of the enzymatic reaction as a function of Fluor-de-lys® concentration at changing fixed concentration of NAD+ (A), and that as a function of NAD+ concentration at changing fixed concentration of the substrate (B) are shown. The double-reciprocal plots of the data of panels (A) and (B) are shown in panels (C) and (D), respectively. The solid smooth lines are the best fit of the data for the sequential two-substrate mechanism using the Grafit software. (TIF) [file pone.0152467.s005.tif]

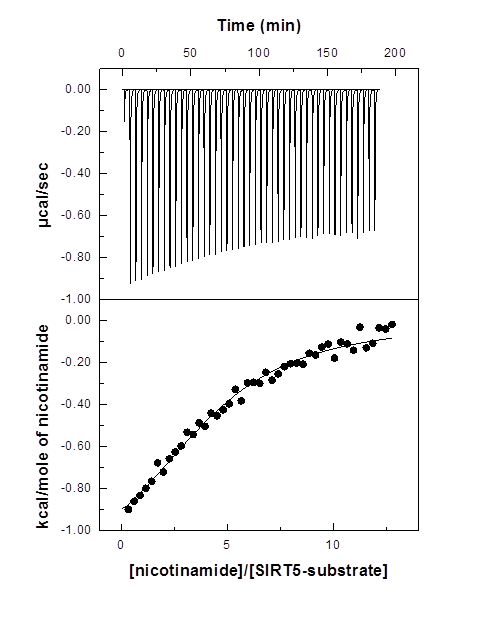

Supplement: S6 Fig — The top panels show the heat signals generated upon titration of 20 μM SIRT5 by 45 injections (5 μl each) of 2 mM nicotinamide in the presence of 1 mM Ac-Suclys-AMC. The area under each peak was integrated and plotted against the molar ratio of nicotinamide to SIRT5. The solid line represent the best fit of the experimental data for the single site binding model of nicotinamide to the enzyme-substrate complex, yielding the magnitudes of Ka and ΔH° as being equal to −(1.3 ± 0.1) kcal/mol and (2.5 ± 0.3) × 104 M-1, respectively. (TIF) [file pone.0152467.s006.tif]

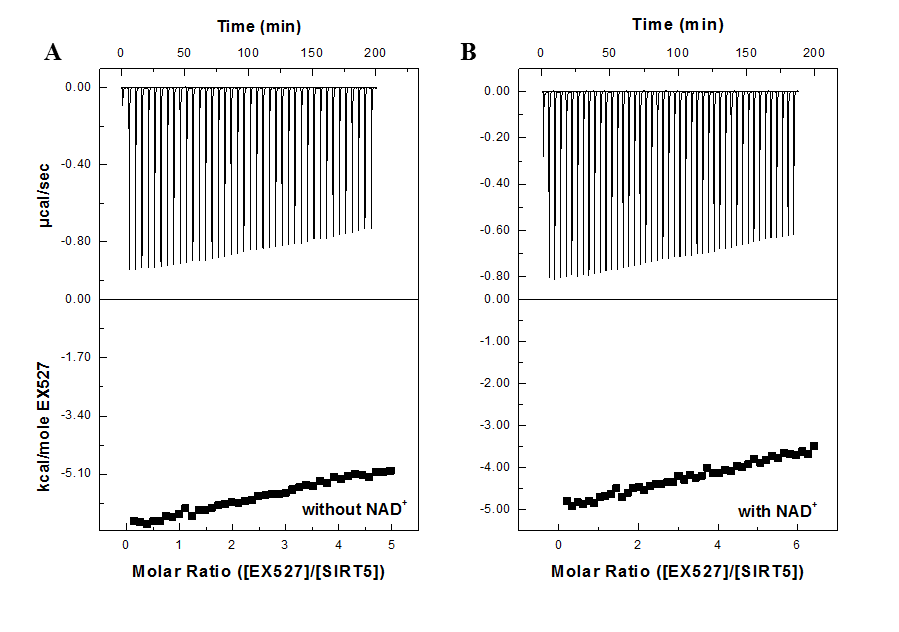

Supplement: S7 Fig — ITC Profile for the Binding of EX527 to SIRT5 in the Absence (A) and Presence of 10 mM NAD+ (B). The top panels show the heat signals generated upon titration of 20 μM SIRT5 Y102A/R105I double mutant by 45 injections (5 μl each) of 500 μM EX527. The area under each peak was integrated and plotted against the molar ratio of EX527 to SIRT5 Y102A/R105I. Due to miniscule change in the heat signals, the ITC data could not be reliably analyzed by any thermodynamic model. (TIF) [file pone.0152467.s007.tif]
